# Reversible Polymer–Protein Functionalization by Stepwise Introduction of Amine-Reactive, Reductive-Responsive Self-Immolative End Groups onto RAFT-Derived Polymers

**DOI:** 10.1021/acsbiomaterials.2c01106

**Published:** 2023-01-25

**Authors:** Maximilian Scherger, Yannick A. Pilger, Patric Komforth, Hans-Joachim Räder, Lutz Nuhn

**Affiliations:** † Max Planck Institute for Polymer Research, Ackermannweg 10, Mainz 55128, Germany; ‡ Chair of Macromolecular Chemistry, Department of Chemistry and Pharmacy, Julius-Maximilians-Universität Würzburg, Röntgenring 11, Würzburg 97070, Germany

**Keywords:** self-immolative linkers, reductive-responsiveness, RAFT-polymer end group, post-polymerization modification, reversible protein functionalization, nanobody

## Abstract

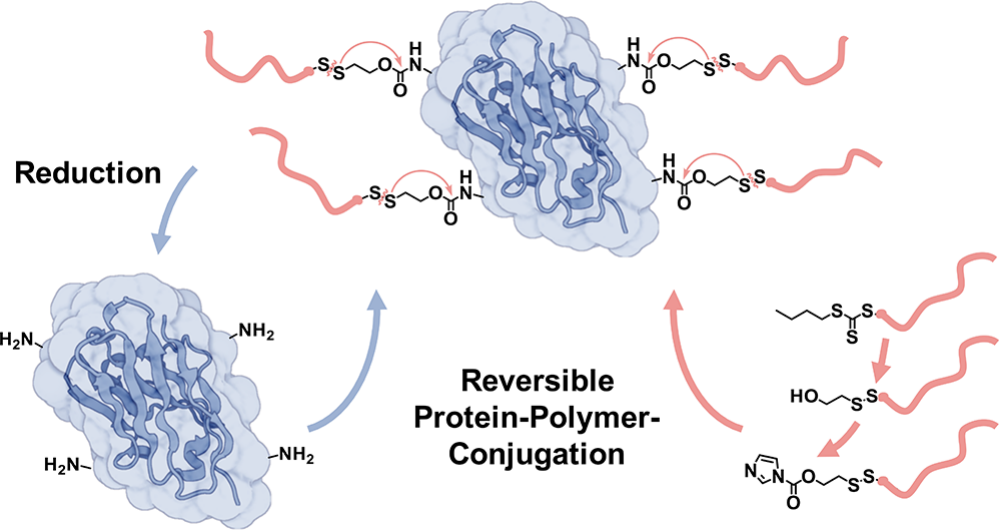

Many promising therapeutic protein or peptide drug candidates are rapidly excreted from an organism due to their small size or their inherent immunogenicity. One way to counteract these effects is PEGylation, in which the biopolymer is shielded by synthetic polymers exploiting their stealth properties. However, these modifications are often accompanied by a reduction in the biological function of the protein. By using responsive moieties that bridge the polymer to the protein, a reversible character is provided to this type of conjugation. In this regard, the reductive-responsive nature of disulfides can be exploited via self-immolative structures for reversible linkage to aminic lysine residues and the N-terminus on the protein surface. They enable a traceless release of the intact protein without any further modification and thus preserve the protein’s bioactivity. In this study, we demonstrate how this chemistry can be made broadly accessible to RAFT-derived water-soluble polymers like poly(*N*,*N*-dimethylacrylamide) (pDMA) as a relevant PEG alternative. A terminal reactive imidazole carbamate with an adjacent self-immolative motif was generated in a gradual manner onto the trithiocarbonate chain transfer moiety of the polymer by first substituting it with a disulfide-bridged alcohol and subsequently converting it into an amine reactive imidazole carbamate. Successful synthesis and complete characterization were demonstrated by NMR, size exclusion chromatography, and mass spectrometry. Finally, two model proteins, lysozyme and a therapeutically relevant nanobody, were functionalized with the generated polymer, which was found to be fully reversible under reductive conditions in the presence of free thiols. This strategy has the potential to extend the generation of reversible reductive-responsive polymer–protein hybrids to the broad field of available functional RAFT-derived polymers.

## Introduction

Antibodies are demonstrating great success as biopharmaceuticals since they bring excellent properties as natural defense proteins due to Fc receptor-mediated mechanisms and, among others, exhibit sufficiently long circulation times in the bloodstream compared to other proteins of this size.^1,2^ However, most other promising protein or peptide drug candidates are eliminated much more rapidly from the body, whether due to their smaller molecular size via the kidney or via further unavoidable receptor interactions.^3^ In addition, their overall integrity *in vivo* is limited, as a variety of these biological components exhibit some degree of immunogenicity.^4^

Conjugation of such biopolymers to synthetic polymers can be very beneficial in this regard by increasing their molecular weight and shielding the protein from recognition from the reticuloendothelial system, thus prolonging the biological stability and half-life.^5,6^ PEGylation, i.e., the covalent attachment of poly(ethylene glycol), has become established and has led to the development of several commercially successful preparations such as PEGylated asparaginase, PEGylated adenosine deaminase, PEGylated interferons, and PEGylated granulocyte colony stimulating factor.^7^

Despite these beneficial properties due to PEGylation, this modification is often accompanied by losses in the biological activity of the proteins.^8^ Furthermore, long-term treatment with these therapeutics can lead to PEG accumulation in the organism or even result in the development of anti-PEG antibodies.^9,10^ For this reason, the generation of new reversible tethering strategies that could counteract these effects and the general development of alternatives to PEGylation are of great importance.^10^

In addition, installing degradable linkers between the protein species and the encapsulating polymer can lead to release of the protein *in vivo* by hydrolysis, enzymatic processing, or by reduction. This approach strikes a balance that does not affect biological activity while improving the pharmacokinetic profile.^11^ Of particular interest are reductive-responsive systems, since these retain their integrity during circulation and are only degraded at their site of action by cellular reducing activities, primarily by the presence of abundant glutathione.^12,13^

Thus, poly(*N*-(2-hydroxypropyl) methacrylamide), for instance, was prepared via reversible addition–fragmentation chain transfer (RAFT) polymerization as a PEG alternative with a midchain pyridyl disulfide functionalized chain transfer agent (CTA), which allowed a reversible protein–polymer conjugation to bovine serum albumin (BSA).^14^ Analogously, degradable PEG-like structures could be conjugated to thiols on the protein surface.^15^ For both variants, as it is often the case in the generation of reversible reductive-responsive protein–polymer conjugates, the presence of available sulfhydryl groups on the protein surface is an essential prerequisite. Therefore, the protein must either carry a nondimerized cysteine in native form, as in the case of BSA,^14,16^ or they are artificially generated by chemical modification,^15^ whereby also biorthogonal click chemistry approaches are very helpful.^17−22^ Another possibility is to install free cysteines by genetic engineering.^23^ However, these procedures are often very complex and ultimately lead to the fact that even after reductive release of the protein, parts of the modification still remain on the protein surface and can restrict its function.

This circumstance can be avoided by the use of so-called self-immolative linkers, which make the responsive disulfide chemistry accessible to nonthiol-containing compounds.^24−26^ These spacers, initiated by an external trigger, can spontaneously decompose in an end-to-end degradation or by a cyclization mechanism, releasing the attached cargo without residues.^27−29^ On this regard, approaches have already been published that used an amine-reactive monomer to generate polymers that could efficiently react with protein surface exposed lysine residues or the N-terminus and thus shield antibodies for intracellular targeting until the adjacent self-immolative moiety restored the intrinsic affinity of the protein at its site of action.^30^

**Figure 1:**
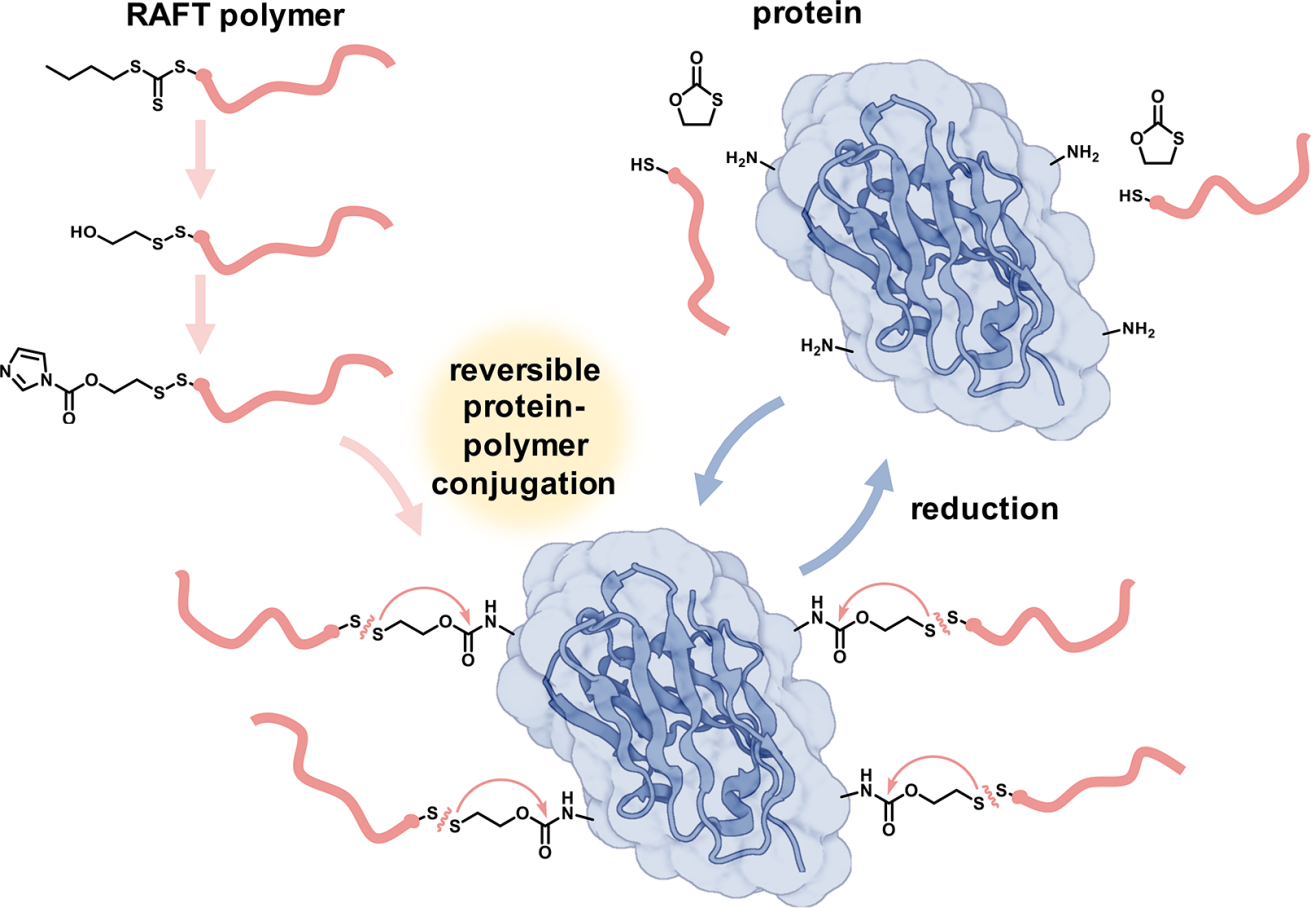
Universal strategy for gradual assembly of reactive imidazole carbamate units with self-immolation motifs at RAFT-derived polymer end groups for reversible protein polymer conjugation.

However, these approaches are severely limited since their applicability is restricted to the utilization of only specific monomers that need to get copolymerized with other functional monomers of interest. Alternatively, highly controlled end group modification reactions can be applied to install amine reactive self-immolative units at the chain end and then reversibly graft them onto proteins.^31^ Interestingly, the trithiocarbonate chain transfer moiety of RAFT-derived water-soluble polymers is well-suited to be gradually converted for that purpose. Taking into account the relevance of pDMA as alternative to FDA-approved PEG (it can, for example, enhance plasma circulation times while circumventing accelerated blood clearance through avoiding IgM responses^32^), and its opportunity to be polymerized under well-defined conditions via RAFT polymerization, it was selected in this study and directly stepwise modified at its RAFT polymerization-derived end group. The resulting polymers provide amine-reactive, reductive-responsive self-immolative end groups to reversibly shield therapeutically relevant proteins (Figure 1). The demonstrated conjugation strategy might be a promising tool to pave the way for extending the generation of reversible reductive-responsive polymer–protein conjugates to a wide variety of accessible multifunctional RAFT polymers.

## Experimental Section

### Materials

Hydrogen peroxide 35% (H_2_O_2_), 2-mercaptoethanol, 4-toluenesulfonyl chloride, potassium bicarbonate, *N*-butylamine, triethylamine (TEA), deuterated solvents, ethyl acetate, cyclohexane, acetonitrile, dimethyl sulfoxide (DMSO), hydrochloric acid (HCl), sodium bicarbonate (NaHCO_3_), 2-bromoethanol, *N,N*-dimethylacrylamide (DMA), 2,2′-azobis(2-methylpropionitrile) (AIBN), bis(pentafluorophenyl) carbonate, 1,8-diazabicyclo[5.4.0]undec-7-en (DBU), and 1,1′-carbonyldiimidazol (CDI) were purchased from Sigma-Aldrich. Potassium sulfite, sulfur, and fluorescamine were purchased from Acros Organics and Alfa Aesar, respectively. *N*,*N*-Dimethylformamide (DMF), chloroform, dichloromethane (DCM), and the BCA (bicinchoninic acid) protein assay kit were obtained from Thermo Fisher Scientific Inc. Methanol (MeOH) and acetone were from Honeywell International. Vivaspin 500 centrifugal concentrators MWCO 10 kDa were purchased from Sartorius.

### Methods

Nuclear magnetic resonance (NMR) spectra were recorded on Bruker Avance 250 and 300 MHz systems. Samples were prepared in deuterated solvents and the recorded spectra were evaluated using MestReNova 14.2.0 from Mestrelab Research.

Size exclusion chromatography (SEC) was conducted on a SECcurity^2^ instrument purchased from PSS, Mainz, equipped with a SECcurity^2^ isocratic pump, a degasser, an auto sampler, an RI detector, a column thermostat and a modified silica gel column (PFG columns, particle size: 7 μm, porosity: 100 Å + 1000 Å) from PSS Polymer Standards Service GmbH. Measurements were conducted at 40 °C with hexafluoro-2-propanol (HFIP) bearing 3 g/L potassium trifluoroacetate as eluent and a flow rate of 0.8 mL/min. Calibration was done with PMMA (PSS Polymer Standards Services GmbH) and elution diagrams were analyzed with PSS WinGPC from PSS Polymer Standard Service GmbH.

Sodium dodecylsulfate polyacrylamide gel electrophoresis (SDS-PAGE) was performed using 16% polyacrylamide gels with a thickness of 0.5 mm loaded with 1 μg of protein which were incubated with 4-fold concentrated Lämmli-buffer (0.2 m Tris-Cl pH 6.8, 8% SDS, 0.4% Bromophenol blue, 40% glycerol). Accordingly, samples were additionally incubated with 1 μL of 2-mercaptoethanol. As a reference, 3 μL of prestained Protein Marker VI (10–245) was loaded to the gel and electrophoreses were performed at 160 V for 1 h and visualization was conducted by Coomassie R-250 staining.

Matrix assisted laser desorption ionization-time of flight mass spectrometry (MALDI-ToF) spectra were conducted using *trans*-2-[3-(4-^*t*^ butylphenyl)-2-methyl-2-propenylidene]malononitrile (DCTB) as matrix on a rapifleX MALDI-ToF/ToF mass spectrometer from Bruker with a 10 kHz scanning smartbeam three-dimensional laser (Nd:YAG at 355 nm) and a 10 bit 5 GHz digitizer in positive ion reflector mode. Data were processed with mMass version 5.5.0 and plotted with GraphPad PRISM version 5.02.

UV–visible spectrophotometry (UV/vis) spectra were recorded using an Agilent Technologies Cary Series UV–vis–NIR spectrophotometer.

Fluorescamine assay and BCA assay data were recorded using a Tecan Spark 20 M microplate reader. For fluorescamine assay, excitation wavelength was set to 390 nm and emission wavelength to 475 nm. BCA assay was conducted using an absorbance wavelength of 562 nm.

### Small Molecule Syntheses

Prior to polymer syntheses and postpolymerization modifications, the chain transfer agents (CTAs) and other building blocks were synthesized as described in the Supporting Information.

### Synthesis of Poly(*N*,*N*-dimethylacrylamide) (pDMA)

pDMA was synthesized as previously reported.^31^

### Synthesis of pDMA-SSC_2_H_4_OH via 2-Mercaptoethanol and O_2_ Oxidation

pDMA (15.0 mg, 6.25 μmol), triethylamine (8.7 μL, 62.5 μmol) and 2-mercaptoethanol (22.0 μL, 312.5 μmol) were dissolved in 0.5 mL of dry DMSO. *N*-Butylamine (6.2 μL, 62.5 μmol) was added and the solution was stirred at room temperature in an open reaction vial for 10 days. The polymer was isolated as a colorless wax by repeated precipitation in diethyl ether in quantitative yields (Supporting Information Figure S1).

### Synthesis of pDMA-SSC_2_H_4_OH via 2-Mercaptoethanol and H_2_O_2_ Oxidation

pDMA (25.0 mg, 10.3 μmol) and *N*-butylamine (5.1 μL, 51.7 μmol) were dissolved in 360 μL of DMSO and stirred for 10 min at room temperature until the yellow color vanished and 2-mercaptoethanol (36.4 μL, 517 μmol) was added. 35% H_2_O_2_-solution in water was added until the H_2_O_2_ content in the reaction mixture reached approximately 10% and it was stirred at room temperature overnight. Solvents were removed under reduced pressure; the residue was taken up in THF and the polymer was isolated by repeated precipitation in diethyl ether as a colorless powder in quantitative yields (Supporting Information Figure S2).

### Synthesis of pDMA-SSC_2_H_4_OH via *S*-(2-Hydroxyethyl) 4-Methylbenzenesulfonothioate

pDMA (10.0 mg, 4 μmol), *N*-butylamine (2.0 μL, 20 μmol), and *S*-(2-hydroxyethyl) 4-methylbenzenesulfonothioate (9.3 mg, 40 μmol) were dissolved in 0.5 mL of dry DMSO and stirred at room temperature for 6 days. The polymer was isolated by repeated precipitation in diethyl ether in quantitative yields (Supporting Information Figure S6 and Figure S7).

### Synthesis of Methyl Poly(*N*,*N*-dimethylacrylamide) (mpDMA)

*N*,*N*–Dimethylacrylamide (519 μL, 500 mg, 5.04 mmol), AIBN (6.6 mg, 0.04 mmol), and methyl 2-(((butylthio) carbonothioyl) thio) propanoate (50.9 mg, 0.20 mmol) were dissolved in 2.5 mL of DMF (2 M DMA) and the reaction mixture was subjected to three freeze–pump–thaw cycles. The solution was stirred at 80 °C for 3 h and mpDMA (250 mg, 45%) was isolated by repeated precipitation in cold diethyl ether (Supporting Information Figures S9–S11).

### Synthesis of mpDMA-SSC_2_H_4_OH via *S*-(2-Hydroxyethyl) 4-Methylbenzenesulfonothioate

mpDMA (100.0 mg, 27 μmol), *N*-butylamine (18.8 μL, 190 μmol), and *S*-(2-hydroxyethyl) 4-methylbenzenesulfonothioate (88.3 mg, 380 μmol) were dissolved in 2.0 mL of dry DMSO and stirred at room temperature for 3 days. Aminolysis of the trithiocarbonate end group was monitored by UV/vis spectroscopy following the absorbance at 310 nm and at 430 nm (Supporting Information Figure S12). The 2-hydroxyethyl disulfide end group modified polymer could be isolated by repetitive precipitation in diethyl ether in quantitative yields (Supporting Information Figures S13–S15).

### Synthesis of mpDMA-SIL-COPFP

mpDMA-SSC_2_H_4_OH (30.0 mg, 8.2 μmol), and bis(pentafluorophenyl) carbonate (22.7 mg, 57.7 μmol) were dissolved in 300 μL of dry THF and 1,8-diazabicyclo[5.4.0]undec-7-en (DBU; 1.72 μL, 11.6 μmol) was added. The solution turned red immediately and was stirred overnight at room temperature. The polymer was isolated by repeated precipitation in diethyl ether in quantitative yields (Supporting Information Figure S16 and Figure S17).

### Synthesis of mpDMA-SIL-IMD

Carbonyldiimidazole (CDI; 12.35 mg, 76.19 μmol) and mpDMA-SS-C_2_H_4-_OH (25 mg, 7.62 μmol) were dissolved in 1 mL of DCM under nitrogen and stirred overnight at room temperature. The polymer was isolated quantitatively by repeated precipitation in diethyl ether in quantitative yields (Supporting Information Figures S18–S20).

### Protein Conjugations

Lysozyme (10 μL of 10 mg/mL in PBS, 100 μg) was added to mpDMA-SIL-IMD (9.3 mg, 450 eq. per protein) in 90 μL of PBS and the solution was incubated overnight at 37 °C. Analogously, α-MMR Nb (73.5 μL of 1.36 mg/mL in PBS) was added to mpDMA-SIL-IMD (9.1 mg, 450 eq. per protein) in 26.5 μL of PBS and the solution was incubated overnight.

Five microliters of the protein–polymer solution were taken out and stored for analysis by SDS-PAGE, while the rest was purified using Vivaspin 500 centrifugal concentrators (molecular weight cutoff 10 kDa). For this, the filters were first washed three times with water. Afterward, the protein solution was added to the centrifugal concentrator, washed three times with water and concentrated for 2 h. The remaining volume was filled up to 95 μL with PBS to afford a 1 mg/mL protein–polymer conjugate solution.

To verify the concentration of the purified protein–polymer solutions, a BCA assay was conducted. For this, nonmodified lysozyme and α-MMR Nb solutions with concentrations ranging from 0 μg/mL to 40 μg/mL were used as sets of proteins standards. An aliquot of the purified polymer protein conjugates (10 μL) was diluted with 490 μL of PBS. One-hundred fifty microliters of each sample solution was then pipetted into a 96 well plate in triplicate. It was mixed with 150 μL of BCA working reagent solution and the microplate was incubated at 37 °C for 2 h. After the microplate cooled down to room temperature, the absorbance at 562 nm was measured. A standard curve was created by plotting each triplicate against its concentration in μg/mL. It was used for calculating the protein concentrations of the protein–polymer conjugate solutions affording quantitative yields by almost full protein recovery. All measurements were carried out in triplicate.

For SDS-PAGE analysis, restoration of native proteins was achieved by reduction of the protein–polymer conjugate. Thus, 2 μL of protein–polymer solution were diluted with 3.5 μL of H_2_O, treated with 2 μL of 2-mercaptoethanol and incubated overnight. The next day the reduced samples as well as the nonreduced samples at same concentrations were mixed with Lämmli buffer, incubated for 5 h, and analyzed by SDS-PAGE.

To determine the degree of functionalization per protein, fluorescamine assays were conducted. The assay relies on the reaction of the free lysine amine and the protein N-terminus with fluorescamine to form a fluorescent product. After the reversible modification of the amine residues with mpDMA, a reduction in fluorescence compared to that of a solution of unmodified protein at the same concentration (determined by BCA assay) can be observed. This fluorescence intensity reduction can be quantified and correlated to the degree of functionalization. Protein standards of lysozyme and α-MMR Nb in PBS were prepared from 0 μg/mL to 20 μg/mL, and the modified protein solutions were investigated at a concentration of 10 μg/mL. A 3 mg/mL fluorescamine stock solution in DMSO was prepared and 3 volume parts of the protein solution were mixed with 1 volume part fluorescamine. The microplate was left to incubate for 15 min protected from light and afterward its fluorescence emission was recorded over time. For restoration of the native proteins, all fluorescamine assay measurements (including each protein calibrations) were additionally conducted in the presence of 10 mM dithiothreitol (DTT). All measurements were further carried out in triplicate.

## Results and Discussion

As previously reported, we were able to substitute the thiocarbonyl moiety of the RAFT polymer end group with a reactive carbonate using a symmetric self-immolative linker approach.^31^ Via this reactive carbonyl and its susceptibility to amines, lysine residues of proteins could be functionalized with polymers. However, this approach is very wasteful in its preparation and less efficient; consequentially, here, we suggest a different route in which the reactive moiety is gradually built up at the polymer end with high integrity.

To this end, in a one-pot reaction, the RAFT end group should first be removed by aminolysis, and the *in situ* generated thiol subsequently be further modified, eventually resulting in an alcohol group bridged via a disulfide at the polymer end. This hydroxy group can in turn be converted into a reactive carbamate.

**Figure 2:**
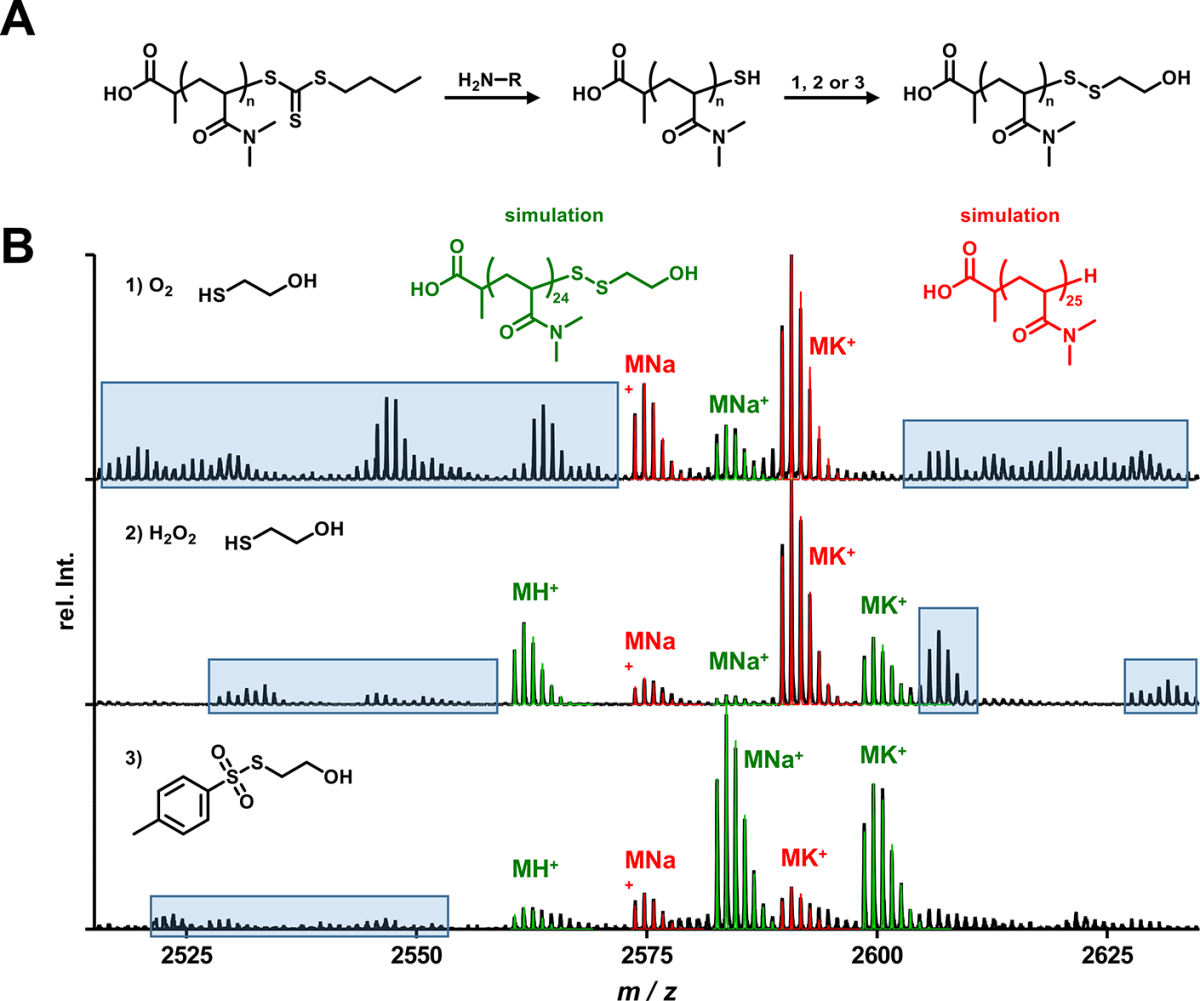
Comparison of different methods to introduce terminal disulfide bridged hydroxy groups on RAFT-derived polymers. (A) General synthesis route. The thiocarbonyl of the RAFT end group is removed by aminolysis, revealing *in situ* a terminal thiol which in turn is consumed in various ways. (B) Comparison of MALDI-ToF analyses of yielded polymers. Desired product species (green) as well as degradation product species (red) due to laser irradiation during MALDI measurement could be observed (1) for the conversion with 2-mercaptoethanol in the presence of O_2_, (2) for the conversion with 2-mercaptoethanol in the presence of H_2_O_2_, and (3) for the conversion with the tosylthiolate derivative. Varying amounts of impurities (blue boxes) were detected.

For the introduction of the alcohol group, three different approaches were investigated, which, however, had in common an initial aminolysis of the thiocarbonyl group of RAFT-derived poly(*N*,*N*-dimethylacrylamide) (pDMA) with *N*-butylamine (Figure 2 and Supporting Information Figures S1–S7). In two approaches, 2-mercaptoethanol was additionally present to provide a disulfide by *in situ* oxidation of the liberated thiol end group. As oxidizing agent, either atmospheric oxygen was applied by stirring the reaction vessel vigorously for several days without sealing or hydrogen peroxide was added. In a third approach, the bromide of bromoethanol was first substituted with tosyl thiolate, resulting in an activated disulfide with a terminal hydroxyl group. This activated disulfide can be substituted in the presence of thiols, such as the *in situ* generated terminal thiol group of the RAFT polymer, in disulfide exchange reactions, which would also result in a disulfide-bridged hydroxy group.

The corresponding reactions were conducted and the end group fidelity was carefully investigated by mass spectrometry (Figure 2 and Supporting Information Figures S1, S2, and S6). First, a species corresponding to the mass of the desired polymer was found overall in each batch (green). In addition, a degradation product resulting from laser irradiation during MALDI measurement could be detected in each sample as well (red).^33−36^ However, the degrees of impurities, or side reactions and byproducts, were different (blue boxes). Despite exhibiting mild reaction conditions and a longer reaction time, the oxidation with atmospheric oxygen yielded the most byproducts, which could not all be identified in detail. If the harsher oxidant hydrogen peroxide was used instead, 2-mercaptoethanol was incorporated much more efficiently onto the polymer and the number of byproducts could be reduced. However, when the activated disulfide route was followed, the undesired byproducts were almost gone (or close to the noise level of the measurement), and thus it is considered the most promising strategy for this setup.

Next, suitable ways to introduce reactive carbonates in the context of protein modifications via lysine side chains were investigated. The literature reports less beneficial properties for trichlorophenyl, trimethylaminophenyl, hexafluoropropanol, and nitrophenyl carbonates.^30^ Dutta et al. were able to demonstrate that the aforementioned groups are hydrolytically stable to some extent but also exhibit poor consumption rates with amines.^30^*N*-Hydroxysuccinimidyl, on the other hand, excels with rapid aminolysis, but this competes with equally rapid hydrolysis. Accordingly, pentafluorophenyl (PFP) carbonates with moderate hydrolytic stability and simultaneous susceptibility to amines showed optimal conditions for the modification of lysine side chains in aqueous medium. They can be introduced onto hydroxy groups by bis(pentafluorophenyl) carbonate (bis-PFP carbonate).

However, since it is also known that bis-PFP carbonate are able to react with carboxylic acids,^37^ which would also lead to a protein–polymer–protein cross-linking/an irreversible amide-based protein–polymer conjugate, we first adapted the CTA used for the polymerization of pDMA. The carboxylic acid of the CTA was esterified with methanol prior to polymerization leading to a carboxylic methyl ester terminated pDMA (mpDMA) (Supporting Information Figures S8–S15). This should ultimately prevent any nucleophilic attack of this group on reactive carbonates in order to minimize the aforementioned side reactions at this chain end of the RAFT-derived polymer.

**Figure 3:**
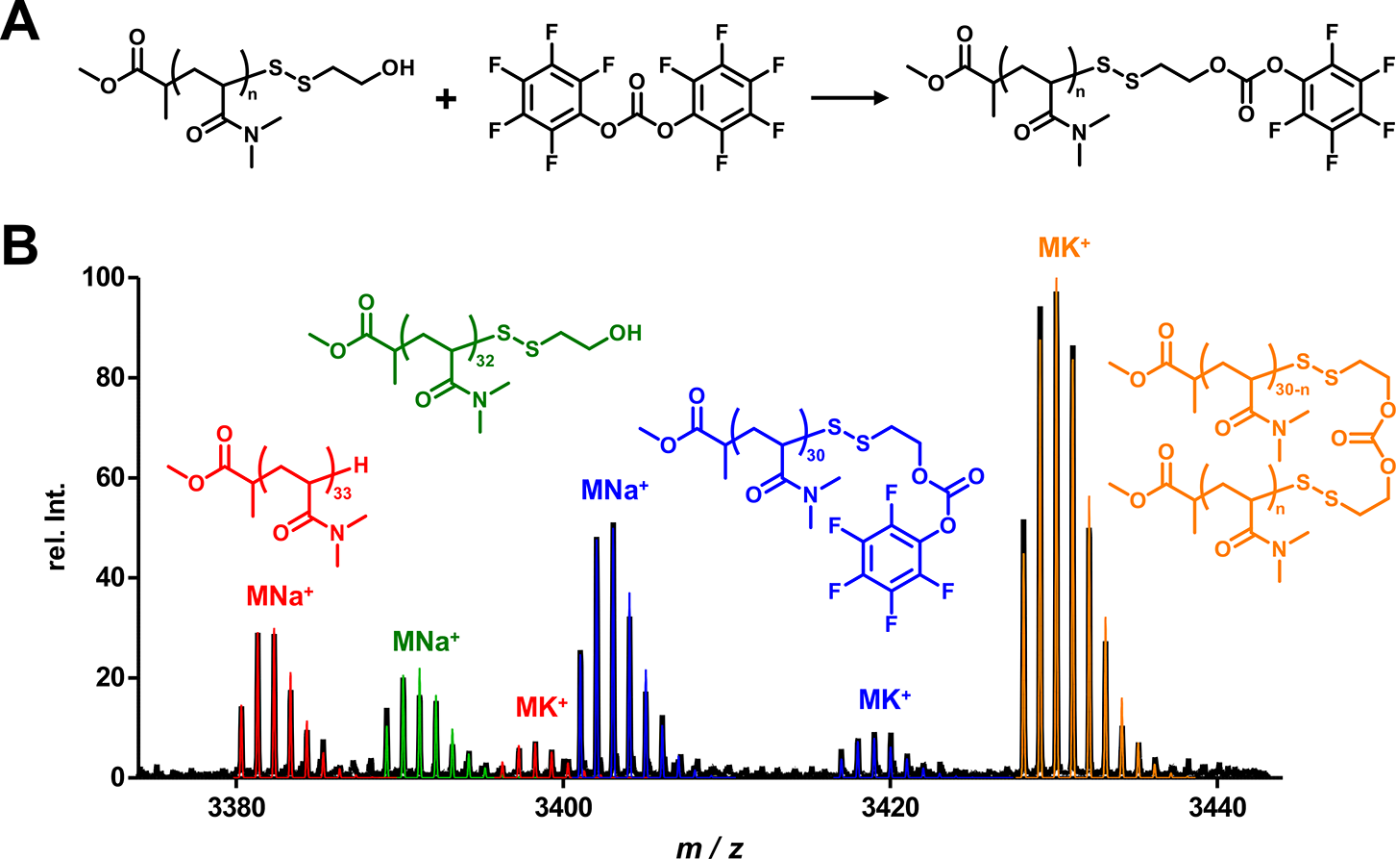
Conversion of mpDMA-SSC_2_H_4_OH with bis-PFP. (A) Reaction scheme. (B) MALDI-ToF analysis. The desired polymer (blue) as well as a degradation product (red) were observed. Additionally, unmodified starting polymer (green) and the formation of polymer dimers (orange) could be demonstrated.

Finally, for the conversion of the terminal hydroxy group, bis-PFP carbonate was used with the aid of an auxiliary base, as already established for the modification of other alcohol end group bearing polymer systems.^38^ The isolated polymer was again analyzed by mass spectrometry (Figure 3 and Supporting Information Figures S16 and S17), and indeed the desired polymer species was identified (blue). The previously observed degradation product could also be detected again here (red). However, in addition, the formation of polymer dimers was observed, which can be explained by an additional substitution of an introduced PFP carbonate by another polymer chain (orange). Moreover, the reaction did not seem to proceed completely, as even despite the use of an auxiliary base, a significant amount of starting polymer still remained detectable (green). This could be due to the previously described stability of PFP carbonates toward OH-nucleophiles. However, assuming the instability of the formed product compound, it can possibly restore the starting compound during the MALDI measurement or even favor the actual reaction with cleavage of the carbonate. Ultimately, no control over this type of end-group functionalization could be gained in the following either by changing the auxiliary base, solvent, temperature, reaction time, or order of addition of the individual components.

Since no reaction conditions could be found under which the generation of a reactive PFP carbonate at the chain end could be implemented in a controlled manner, but the desired product was nevertheless partially detectable along with some byproducts, alternative reactive carbonate reagents were investigated. Among them, carbonyldiimidazole (CDI) was chosen, since at least a few papers were also published using this component on the topic of protein modification.^39,40^

Again, a terminal alcohol was first introduced via the tosylthiolate starting from mpDMA after aminolysis of the thiocarbonyl unit. The polymer mpDMA-SSC_2_H_4_OH was then treated with CDI, whereby an imidazole residue was substituted by the hydroxy group of the polymer. The released imidazole subsequently acts itself as an auxiliary base for further reactions. It eliminates the need for additional application of such reagents for this conversion and offers greater reaction control, especially during the early stage of the reaction. This approach enables a gradual formation of RAFT-derived polymers whose terminal thiol-containing moiety are extended by a disulfide in whose vicinity a reactive imidazocarbamate is localized (mpDMA-SIL-IMD).

**Figure 4:**
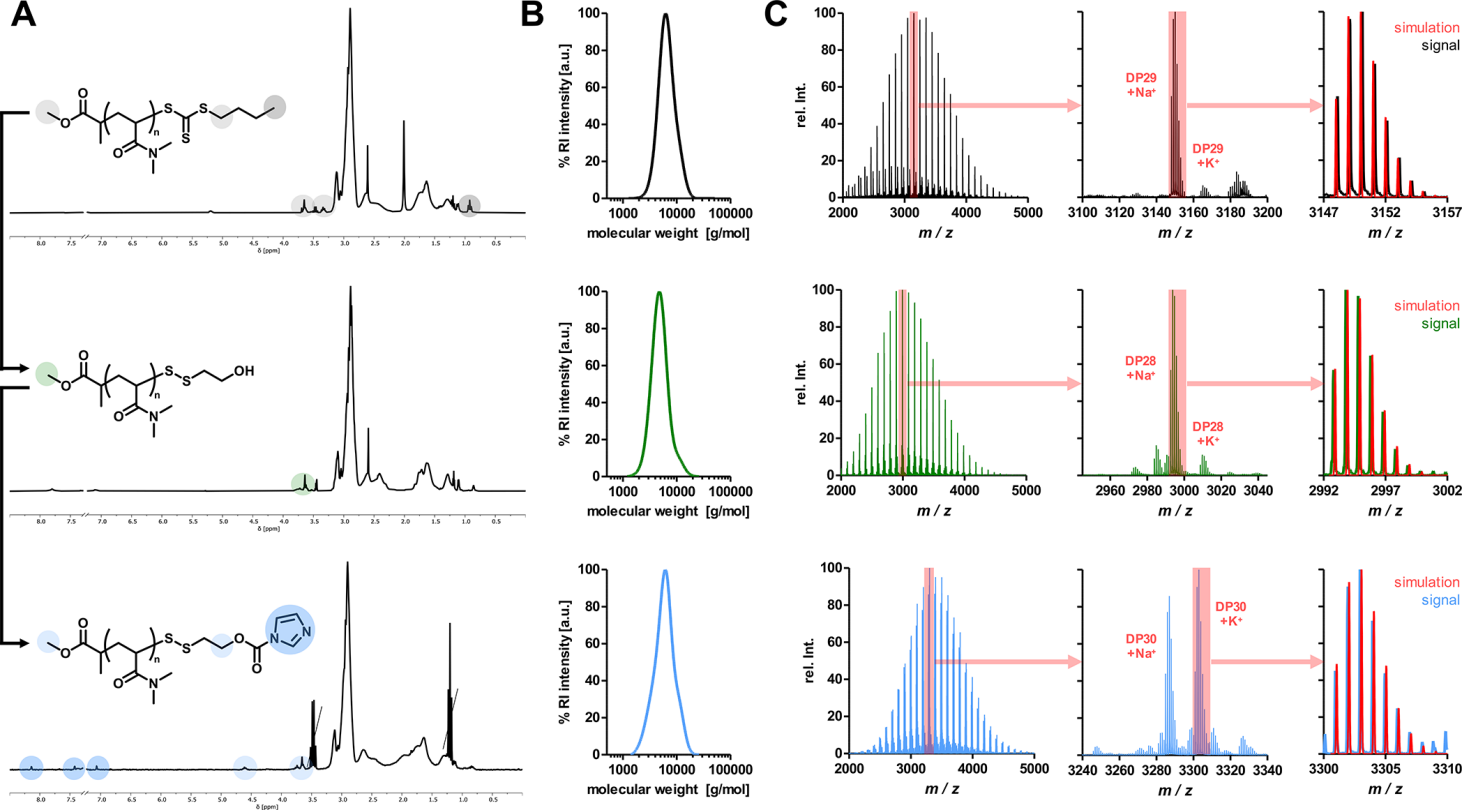
Polymer characterization of mpDMA (black), mpDMA-SSC_2_H_4_OH (green), and mpDMA-SIL-IMD (blue) for the gradually formation of self-immolation motif-terminated RAFT-derived polymers. (A) ^1^H NMR spectra. The vanished RAFT end group proton signals and introduced aromatic and adjacent carbamate proton signals are highlighted. (B) SEC traces exhibiting monomodal molecular weight distributions. (C): MALDI-ToF MS data: full polymer mass range (left); zoomed mass range of DP with highest relative intensity (middle); overlay of DP with highest relative intensity and its corresponding simulated isotope pattern in red (right).

The progress of each step of the synthesis was followed by NMR, size exclusion chromatography (SEC), and mass spectrometry (Figure 4). In the ^1^H NMR spectra (Figure 4A and Supporting Information Figure S18), the successful removal of the trithiocarbonyl moiety was first observed, as the signal from the protons of the adjacent methylene group disappeared from mpDMA (gray) to mpDMA-SSC_2_H_4_OH (green). In addition, binding of the disulfide-bridged imidazocarbamate was evidenced by the appearance of both aromatic imidazole proton signals and proton signals from the methylene group adjacent to the carbamate (blue). The actual binding was further confirmed by a DOSY experiment, where the aforementioned proton signals exhibit similar diffusion properties as the proton signals of the polymer backbone (see Supporting Information Figure S19). The SEC traces demonstrated narrow monomodal distributions for all three polymers (Figure 4B). The average molecular weight distribution of mpDMA-SSC_2_H_4_OH appeared slightly smaller compared to the starting polymer, although the actual molecular weight should have changed only marginally. This could be due to the change in polarity by replacing an apolar terminal alkyl chain by a polar alcohol and thus influencing the hydrodynamic radius of the polymer. Finally, mass spectrometry indeed indicated very high end-group fidelity for all steps of this synthetic route (Figure 4C). In each case, the signals of the main species were in strong agreement with the corresponding simulations, once again convincingly demonstrating a successful gradual generation of mpDMA-SIL-IMD bearing a lysine reactive self-immolation motif.

**Figure 5:**
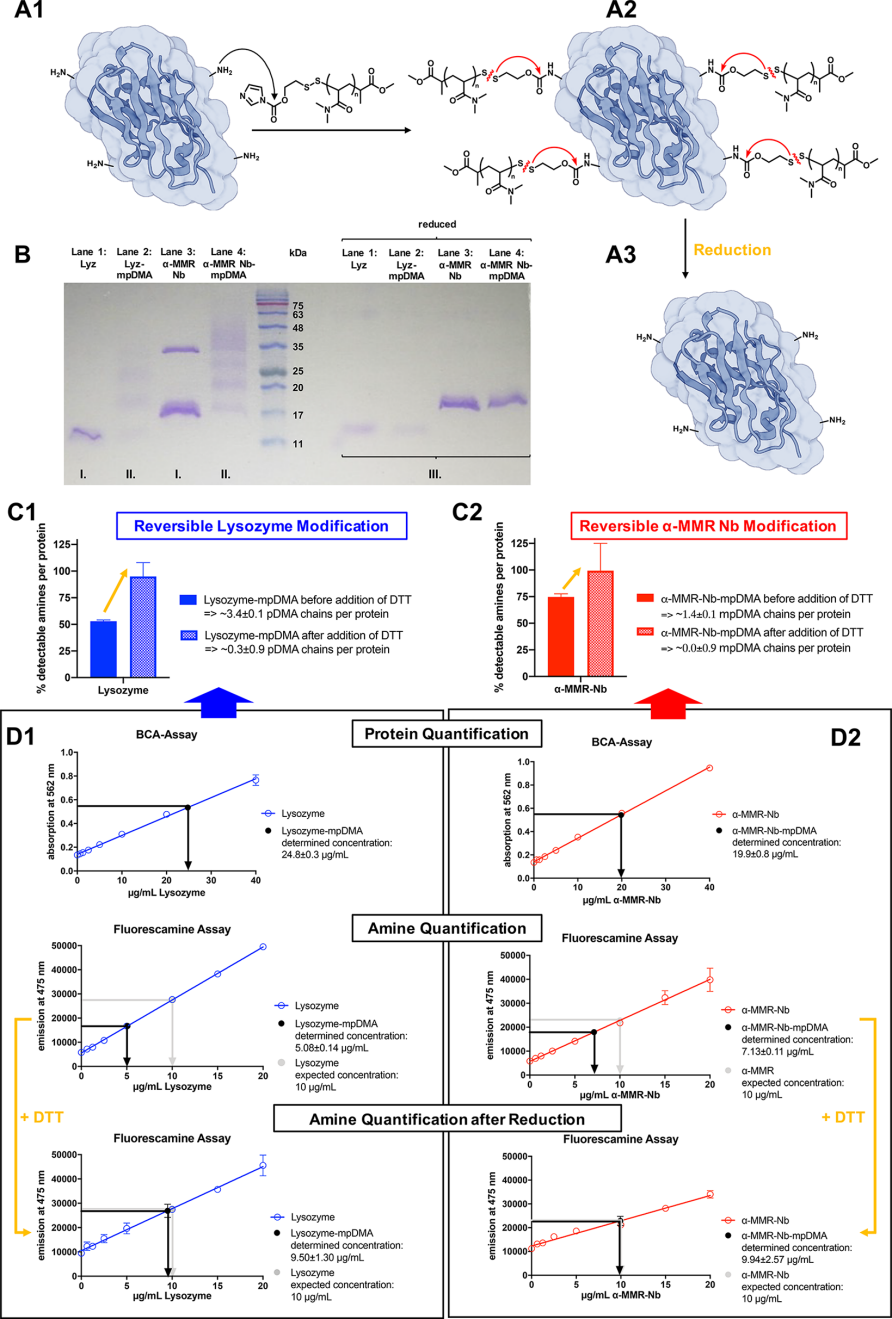
Reversible mpDMA conjugation of Lyz and α-MMR Nb. (A) The schematic illustration shows the synthetic conjugation of mpDMA-SIL-IMD on primary amines of protein surface exposed lysines and the N-terminus (A1 to A2) and its subsequent reversibility due to reduction and occurring self-immolation (A2 to A3). (B) The SDS-PAGE shows both native Lyz (line 1) and native α-MMR Nb (line 3) and the corresponding protein–polymer conjugates (Lyz-mpDMA, line 2; α-MMR Nb-mpDMA, line 4) with and without incubation with 2-mercaptoethanol. 5IVO was used as representative nanobody structure and processed with BioRender.com. (C) The percentage of detectable amines per protein was determined for lysozyme (C1) and α-MMR Nb (C2) before and after reduction by 10 mM DTT to estimate the reversible conjugation of about 3–4 pDMA chains to lysozyme and 1–2 pDMA chains to α-MMR Nb. (D) To calculate these values, first BCA assays of the purified polymer–protein conjugates were performed to quantify the amount of protein, followed by fluorescamine assays to detect the number of amines per protein before and after incubation with 10 mM DTT for 1 h. Both measurements could successfully be recorded for lysozyme (D1) and α-MMR Nb (D2).

The well-defined polymers generated by this procedure were then investigated not only for their ability to conjugate to proteins via the reactive carbamate. Special interest was put into whether this type of modification is reversible and can be initiated by an external trigger into a self-immolation process. For this purpose, lysozyme (Lyz) as model protein and a therapeutically more relevant nanobody against the mannose receptor of macrophages (α-MMR Nb) (overexpressed on protumoral macrophages)^41^ were used to further elucidate this issue. These proteins (I, Figure 5A) were treated in physiological PBS medium with an excess of mpDMA-SIL-IMD, whereby the primary amines of the lysine side chains as well as the protein’s N-terminus can undergo nucleophilic substitution of the reactive carbamates, thus covering the surface of the biopolymers with a synthetic polymer corona (II, Figure 5A). After protein–polymer conjugation the modified protein can be purified from excess polymer by spin filtration with a molecular weight cutoff of 10 kDa, as polymers with molecular weights below 5 kDa are used for the reversible conjugation. Under reducing conditions, the disulfides should get cleaved and the liberated thiols trigger a linker self-immolation, resulting in restoration of the native proteins (III, Figure 5A).

The individual steps of this experiment were followed by sodium dodecyl sulfate polyacrylamide gel electrophoresis (SDS-PAGE), and both native Lyz (line 1) and native α-MMR Nb (line 3) and the corresponding protein–polymer conjugates (Lyz-mpDMA, line 2; α-MMR Nb-mpDMA, line 4) were applied with and without incubation with reducing agent (Figure 5B). The native proteins initially showed only their distinct bands according to their molecular weight, although it should be noted that α-MMR Nb was present as both monomer and dimer due to the presence of a genetically engineered C-terminal cysteine. Upon conjugation, these bands disappeared and were replaced by diffuse but still slightly visible shadows explained by decoration of the proteins with different numbers of polymer chains of various lengths, resulting in a highly heterogeneous molecular weight distribution. No difference was observed for the modified proteins appearing as diffuse shadows in the reaction mixture as well as after purification by spin filtration on the SDS PAGE (compare Supporting Information Figure S21). However, when these samples were incubated with intracellular physiological concentrations of free thiols by adding 2-mercaptoethanol in the millimolar range before application to the gel, the disulfide bridges of the polymer conjugation were reduced and cleaved. This finding was supported by the fact that here bands of modified proteins again showed only one distinct band, which exhibited identical migration behavior in the gel as the reduced native proteins (Figure 5B, as well as Supporting Information Figure S22 for the purified protein conjugation samples). It should be noted that sufficient space was inserted between untreated and reduced samples on the SDS gel, since 2-mercaptoethanol was able to diffuse back and forth between the individual lines and otherwise also reduced untreated samples during electrophoresis.

To further quantify the number of polymers that could be reversibly conjugated to the two proteins (Figure 5C), the isolated polymer–protein conjugates were first quantified by BCA assay to determine the amount of isolated protein. Interestingly, this was for both proteins in the expected range of the initially applied amounts of proteins affording quantitative recovery after spin filtration (Figure 5D).

Due to the covalent conjugation of the polymers to the primary amines of the lysine residues or the N-terminus, fluorescamine assays were next conducted. They are sensitive for quantifying the number of primary amines for each protein. Compared to a calibration with the unmodified proteins, a reduction of the detectable amines was recorded by ∼50% for lysozyme and ∼25% for α-MMR Nb (Figure 5C, D). As the lysozyme is equipped with 6 lysines plus one N-terminus, this results in an average of 3–4 pDMA polymer chains per protein, while for the α-MMR nanobody bearing 4 lysines plus one N-terminus, only 1–2 pDMA polymer chains per protein can be estimated (Figure 5C).

Interestingly, when these fluorescamine assays were recorded for both modified proteins in the presence of 10 mM dithiothreitol (DTT), a complete restoration of the unmodified proteins could be observed after 1 h, confirming the self-immolative release mechanism and the full liberation of all amine residues (Figure 5D). Thereby, a kinetic delay was observed for the liberation of the native α-MMR nanobody when the fluorescamine assays were recorded over time after addition of DTT, while for lysozyme, a quantitative release was found immediately (Supporting Information Figure S23 and Figure S24). Consequently, one may speculate on the vast accessibility of primary amines on the protein surface of the lysozyme, which allows for both a better polymer conjugation as well as a rapid reductive responsive release. For the α-MMR nanobody, however, certain restrictions may occur since fewer polymer chains could be conjugated and they were released more slowly.

It is noteworthy that the self-immolatively linked polymer protein conjugates remain stable in PBS over a prolonged time and did not alter the size distribution of the proteins either (Supporting Information Figures S23–S25).

Overall, these experiments demonstrated that the previously prepared polymer mpDMA-SIL-IMD is capable of decorating the surface of proteins and that this modification is reversible by an external trigger in a self-immolation process.

## Conclusion

In this manuscript, a universal strategy is introduced to gradually convert the trithiocarbonate end group of RAFT-polymerized poly(*N*,*N*-dimethylacrylamide) (pDMA) into an amine reactive imidazole carbamate unit with a self-immolation behavior. Three ways to replace the RAFT end group of polymers with a disulfide-bridged alcohol in a one-pot reaction were initially investigated. Although the product was detectable in each case, oxidation with atmospheric oxygen in the presence of 2-mercaptoethanol after aminolysis revealed many side products during MALDI mass spectrometry measurements, which could be decreased by replacing the oxidant with hydrogen peroxide. However, when the alcohol was introduced by an activated disulfide in a disulfide exchange reaction with a tosylthiolate derivative, these conditions allowed a quantitative introduction of the desired end group, as confirmed by MALDI mass spectrometry.

Subsequent reaction with a pentafluorophenyl biscarbonate also led to the formation of an amine reactive carbonate at the polymer end, but control over this reaction could not be gained and many side reactions could not be avoided. A switch to carbonyldiimidazole was finally able to circumvent these issues and the gradual assembly of a reactive imidazole carbamate moiety with a self-immolation motif at the terminus of a RAFT-derived polymer could be obtained. Successful synthesis could be tracked by NMR spectroscopy, size exclusion chromatography, and mass spectrometry.

Finally, two model proteins, lysozyme and a nanobody against the mannose receptor of macrophages, were decorated with this polymer under physiological conditions by reaction with primary amine bearing lysine residues or the N-terminus on their surface. After exposure to endogenous reducing conditions, these modifications were reversed by self-immolation processes and the native proteins were restored, which could be demonstrated by SDS-PAGE as well as fluorescamine assays in combination with BCA assays and thus opens the door to opportunities of various different reversible reductive-responsive polymer–protein hybrids far from conventional PEGylation.

## Supporting Information Available

Experimental data for small molecule syntheses including chain transfer agents, preparation of postpolymerization building blocks, additional data on polymer characterization, NMR spectra and assignments, SEC data and traces, DOSY spectra, SDS-PAGE data, UV/vis data, DLS size distribution data, etc. (PDF)
