# Supplementary material for: Reversible Polymer–Protein Functionalization by Stepwise Introduction of Amine-Reactive, Reductive-Responsive Self-Immolative End Groups onto RAFT-Derived Polymers
Source: ACS Biomater Sci Eng. 2023 Jan 25;10(1):129–38. doi: 10.1021/acsbiomaterials.2c01106 (PMC10777346; doi:10.1021/acsbiomaterials.2c01106)
Supplement: Supplementary file 1 — ab2c01106_si_001.pdf [file ab2c01106_si_001.pdf]

Reversible Polymer-Protein Functionalization by Step-Wise Introduction of Amine-reactive, Reductive-Responsive Self-Immolative End Groups onto RAFT-derived Polymers

-Supporting Information-

Maximilian Scherger,[a] Yannick A. Pilger,[a,b] Patric Komforth,[a] Hans-Joachim Räder,[a] and Lutz Nuhn[a,b]*

[a]: Max Planck Institute for Polymer Research, Ackermannweg 10, 55128 Mainz, Germany

E-mail: lutz.nuhn@mpip-mainz.mpg.de

[b]: Chair of Macromolecular Chemistry, Department of Chemistry and Pharmacy, Julius-Maximilians-Universität Würzburg, Röntgenring 11, 97070 Würzburg

E-mail: lutz.nuhn@uni-wuerzburg.de

*: corresponding author: Prof. Dr. Lutz Nuhn

(lutz.nuhn@mpip-mainz.mpg.de or lutz.nuhn@uni-wuerzburg.de)

2-(butylthiocarbonothioylthio)propanoic acid (PABTC) and poly(*N*,*N*-dimethylacrylamide) (pDMA)

The chain transfer agent 2-(butylthiocarbonothioylthio)propanoic acid (PABTC) was synthesized as previously reported as well as the preparation of poly(*N*,*N*-dimethylacrylamide) (pDMA).[1]

pDMA-SSC_2H_4OH via 2-mercaptoethanol and O_2 oxidation

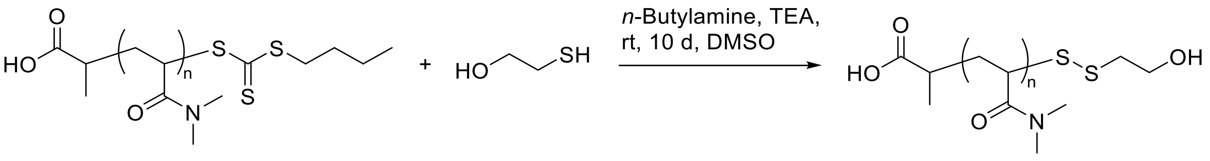

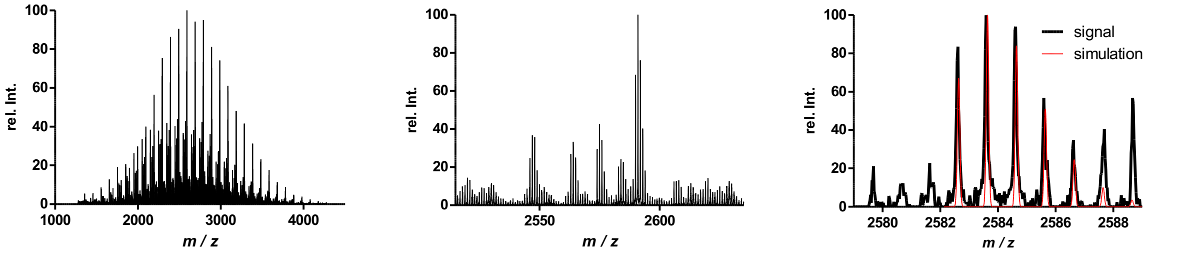

Figure S1: MALDI-ToF MS data of pDMA-SSC_2H_4OH via 2-mercaptoethanol and O_2 oxidation. Full polymer mass range (left); zoomed mass range of DP with highest relative intensity and impurities (middle); overlay of DP with highest relative intensity and its corresponding simulated isotope pattern (MNa^+; right).

pDMA-SSC_2H_4OH via 2-mercaptoethanol and H_2O_2 oxidation

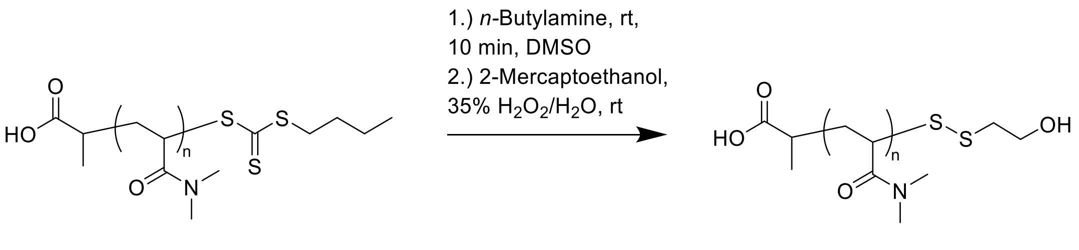

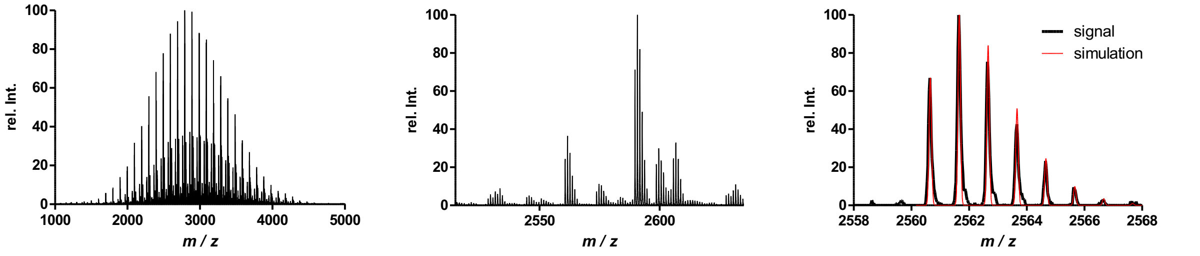

Figure S2: MALDI-ToF MS data of pDMA-SSC_2H_4OH via 2-mercaptoethanol and H_2O_2 oxidation. Full polymer mass range (left); zoomed mass range of DP with highest relative intensity and impurities (middle); overlay of DP with highest relative intensity and its corresponding simulated isotope pattern (MH^+; right).

Potassium 4-methylbenzenesulfinate

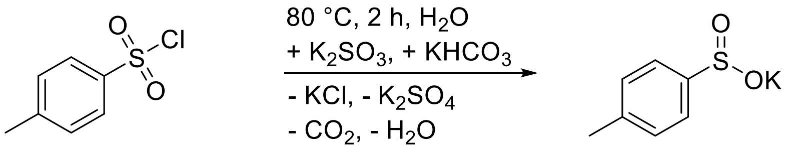

4-toluenesulfonyl chloride (28.59 g, 150 mmol) was added in small portions to a solution of potassium bicarbonate (30.03 g, 300 mmol) and potassium sulfite (79.14 g, 450 mmol) in water (118 mL) at 80 °C. The mixture was further stirred at the same temperature for 2 h, afterwards water was removed in vacuo at 60 °C. The resulting solid was suspended in 300 mL methanol and filtered. Evaporation of methanol gave potassium 4-methylbenzenesulfinate (26.44 g, 136.10 mmol, 91%) as a colorless solid.

^{1}H NMR (300 MHz, D_2O): δ [ppm] = 7.54 (d, J = 6 Hz, 2H, Ar-*H*); 7.37 (d, J = 6 Hz, 2H, Ar-*H*); 2.39 (s, 3H, -CH_3).

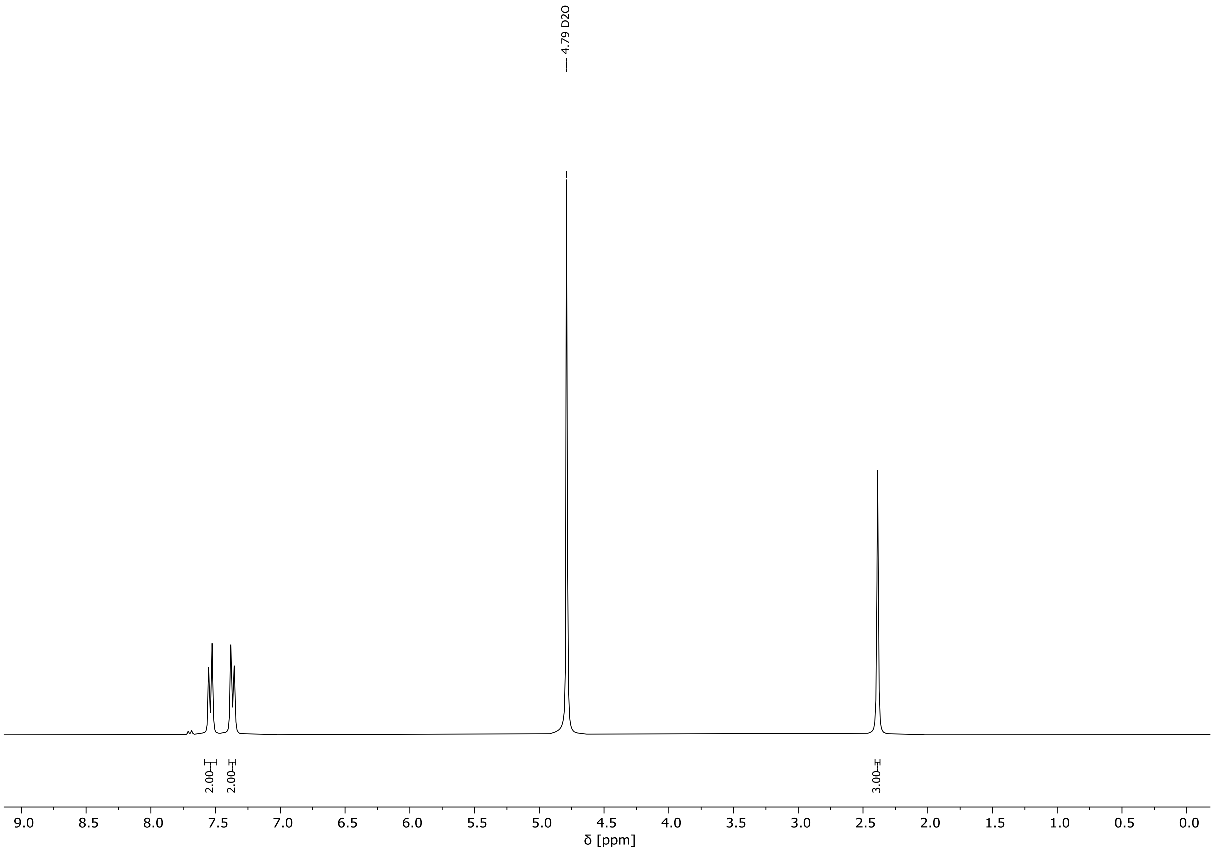

Figure S3: ^{1}H NMR (300 MHz, D_2O) of potassium 4-methylbenzenesulfinate.

Potassium 4-methylbenzenesulfonothioate

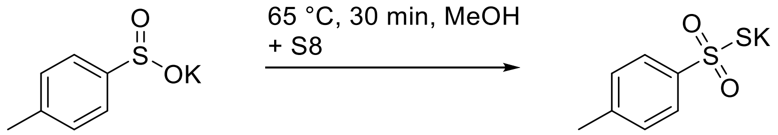

Potassium 4-methylbenzenesulfinate (10.33 g, 50 mmol, 1.00 eq) and sulfur (1.57 g, 49 mmol, 0.98 eq) were suspended in methanol (300 mL). The yellow suspension was refluxed for 30 min to a clear solution, cooled down and evaporated. The colorless residue was triturated with ethanol and again evaporated to yield potassium 4-methylbenzenesulfonothioate (11.30 g, 48.44 mmol, 97%) as a colorless solid.

^{1}H NMR (300 MHz, D_2O): δ [ppm] = 7.82 (d, J = 9 Hz, 2H, Ar-H); 7.38 (d, J = 9 Hz, 2H, Ar-H); 2.41 (s, 3H, -CH_3).

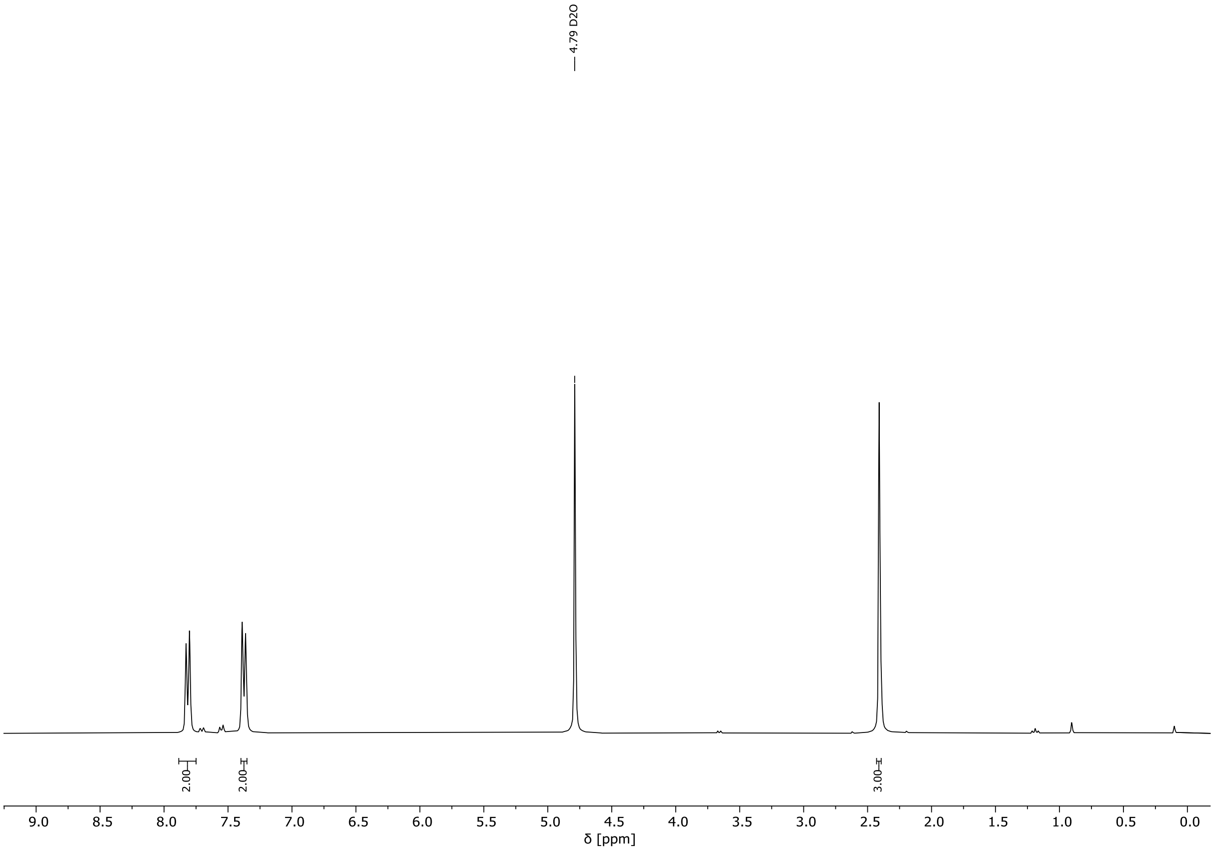

Figure S4: ^{1}H NMR (300 MHz, D_2O) of potassium 4-methylbenzenesulfonothioate.

_S_-(2-hydroxyethyl) 4-methylbenzenesulfonothioate

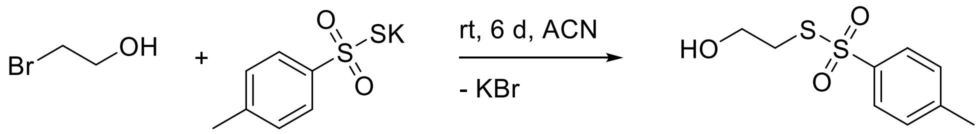

2-Bromoethanol (1.5 g, 12.0 mmol) and 4-methylbenzenesulfonothioate (3.0 g, 13.2 mmol) were stirred in 70 mL dry acetonitrile at room temperature for 6 days. The solvent was removed under reduced pressure, salts were precipitated in acetone and the solution was filtered. Acetone was separated under reduced pressure again and the product was isolated as a colorless oil (2.3 g, 9.90 mol, 83%) and used without any further purification.

R_f=0.16 (cyclohexane/ethyl acetate = 3/1)

^{1}H NMR (300 MHz, $CDCl_3$): δ [ppm] = 7.82 (d, J = 8.1 Hz, 2H, –SO_2–C=C*H*–), 7.35 (d, J = 8.1 Hz, 2H, –C*H*=C–CH_3), 3.85 (t, J = 5.9 Hz, 2H, HO–CH_2–), 3.16 (t, J = 5.9 Hz, 2H, –CH_2–S–), 2.45 (s, 3H, –CH_3).

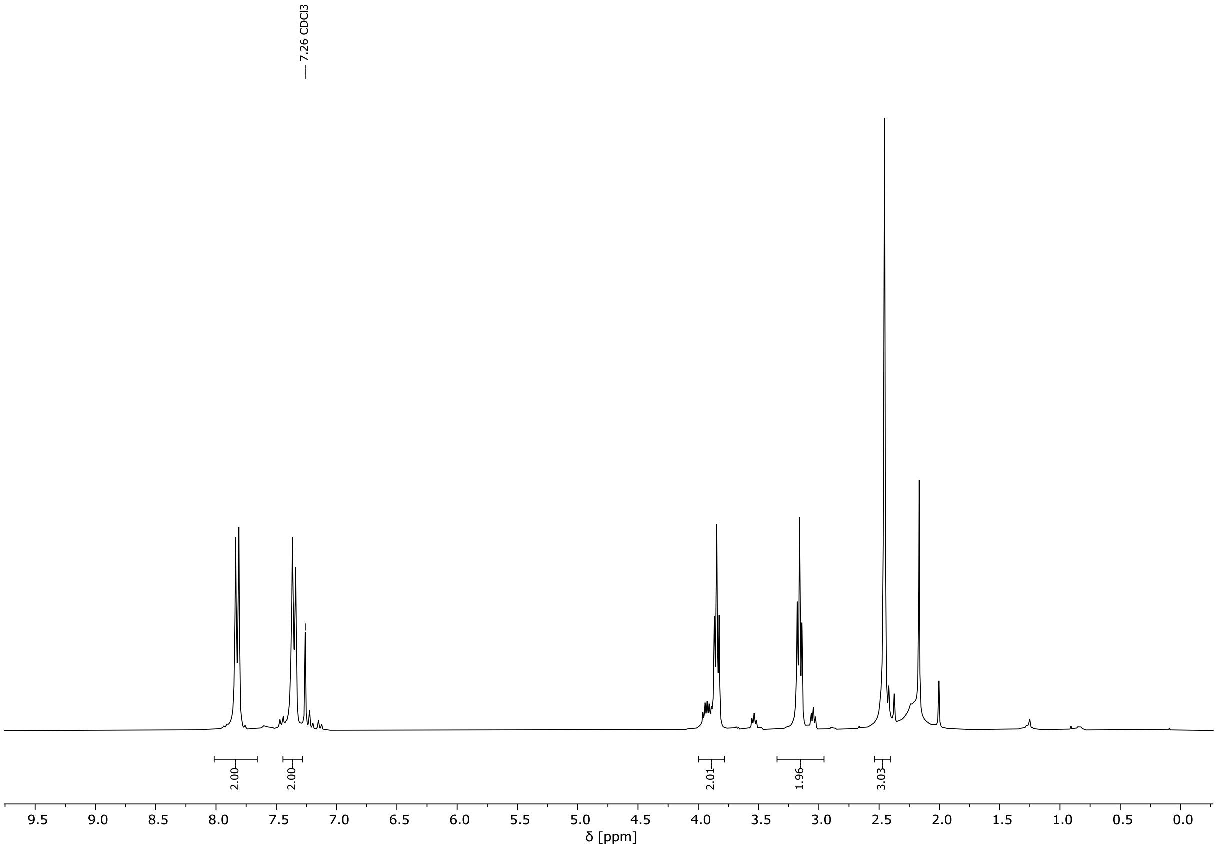

Figure S5: ^{1}H NMR (300 MHz, $CDCl_3$) spectrum of *S*-(2-hydroxyethyl) 4-methylbenzenesulfonothioate.

pDMA-SSC_2H_4OH via *S*-(2-hydroxyethyl) 4-methylbenzenesulfonothioate

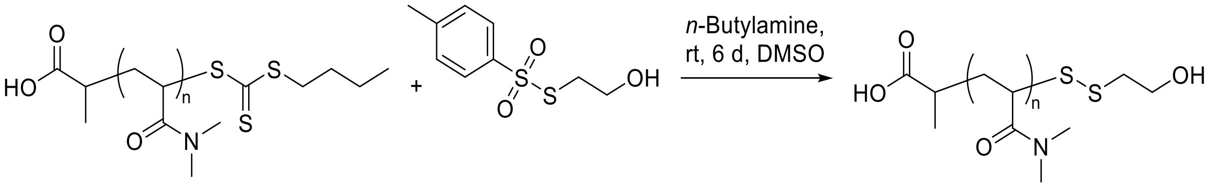

SEC (HFIP; PMMA standard): M_n = 3,450 g mol^{-1}; M_w = 4,024 g mol^{-1}; PDI = 1.17.

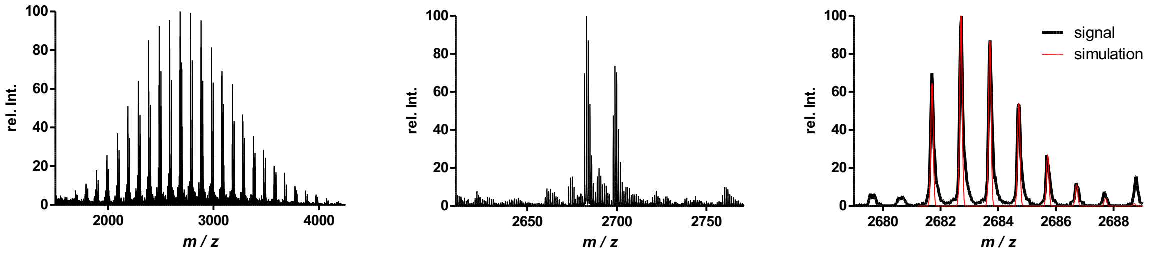

Figure S6: MALDI-ToF MS data of pDMA-SSC_2H_4OH via *S*-(2-hydroxyethyl) 4-methylbenzene-sulfonothioate. Full polymer mass range (left); zoomed mass range of DP with highest relative intensity (middle); overlay of DP with highest relative intensity and its corresponding simulated isotope pattern (MNa^+; right).

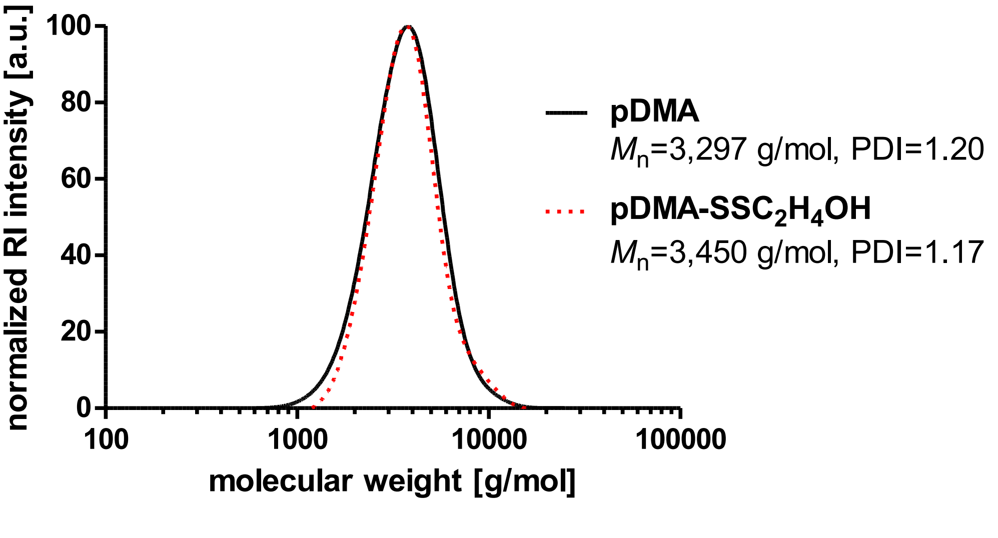

Figure S7: SEC traces of pDMA and pDMA-SSC_2H_4OH with HFIP as eluent.

Methyl 2-(((butylthio)carbonothioyl)thio)propanoate

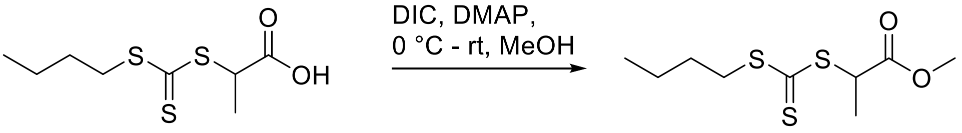

2-(((butylthio)carbonothioyl)thio)propanoic acid (200.0 mg, 0.84 mmol) and 4-dimethylaminopyridine (10.3 mg, 0.08 mmol) were dissolved in 10 mL MeOH in an ice-water bath. *N*,*N'*-Diisopropylcarbodiimide (127.1 mg, 1.01 mmol) in 1 mL MeOH was added dropwise over 30 min. The ice-water bath was removed and the reaction mixture was stirred over the weekend. The solvent was removed under reduced pressure and the residue was taken up in DCM and filtrated. The organic phase was extracted with 1 N HCl and saturated $NaHCO_3$, dried and the solvent was removed. The residue was once again taken up in cold *n*-hexane and filtrated again. Column chromatography in chloroform gave the pure product as a yellow oil (175.2 mg, 0.69 mmol, 83%).

R_f=0.71 (chloroform)

^{1}H NMR (300 MHz, $CDCl_3$): δ [ppm] = 4.84 (q, J = 7.3 Hz, 1H, H_3C–CH–), 3.74 (s, 3H, –O–CH_3), 3.36 (t, J = 7.4 Hz, 2H, –S–CH_2–), 1.74 – 1.63 (m, 2H, –S–CH_2–CH_2–), 1.60 (d, J = 7.4 Hz, 3H, H_3C–CH–), 1.50 – 1.35 (m, 2H, H_3C–CH_2–), 0.93 (t, J = 7.3 Hz, 3H, H_3C–CH_2–).

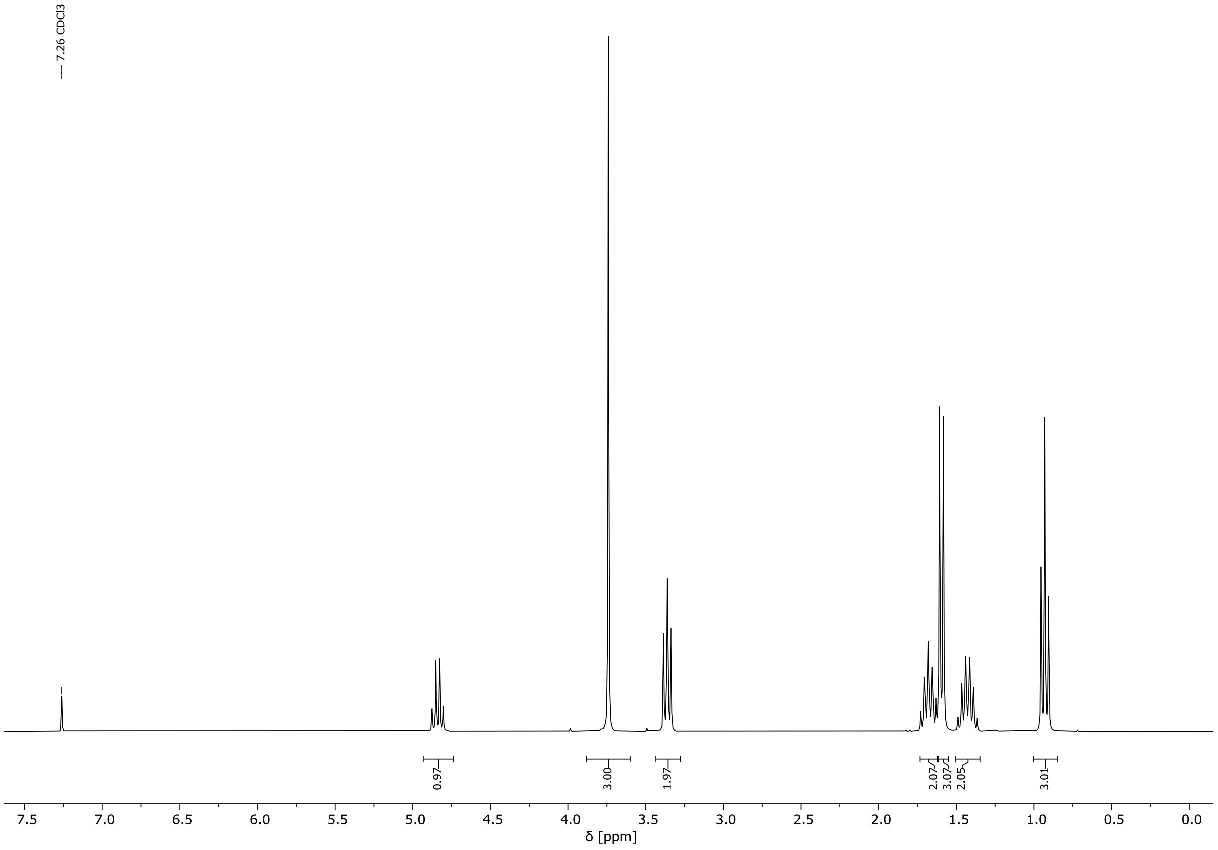

Figure S8: ^{1}H NMR (300 MHz, $CDCl_3$) spectrum of methyl 2-(((butylthio)carbonothioyl)thio)propanoate.

Methyl poly(*N*,*N*-dimethylacrylamide) (mpDMA)

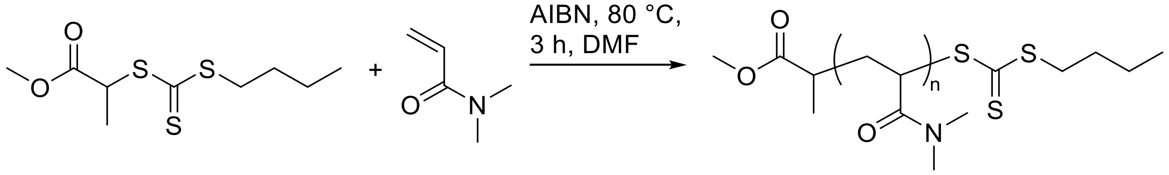

SEC (HFIP; PMMA standard): M_n = 4,411 g mol^{-1}; M_w = 5,021 g mol^{-1}; PDI = 1.14.

^{1}H NMR (300 MHz, $CDCl_3$): δ [ppm] = 5.30 – 5.08 (m, 1H, –S–C*H*–CON(CH_3)$_2$), 3.71 – 3.56 (m, 3H, *H*$_3$C–O–), 3.34 (t, *J* = 7.3 Hz, 2H, –S–C*H*$_2$–CH_2–), 3.19 – 2.76 (m, 210H, –N–(C*H*$_3$)$_2$), 2.75 – 2.26 (m, 35H, –C*H*–CON(CH_3)$_2$), 2.01 – 1.16 (m, 74H, –C*H*$_2$–CH–$CONR_2$ and –S–CH_2–C_2*H*$_4$–), 1.12 (d, *J* = 6.8 Hz, 3H, –CH–C*H*$_3$), 0.92 (t, *J* = 2.3 Hz, 3H, –CH_2–C*H*$_3$).

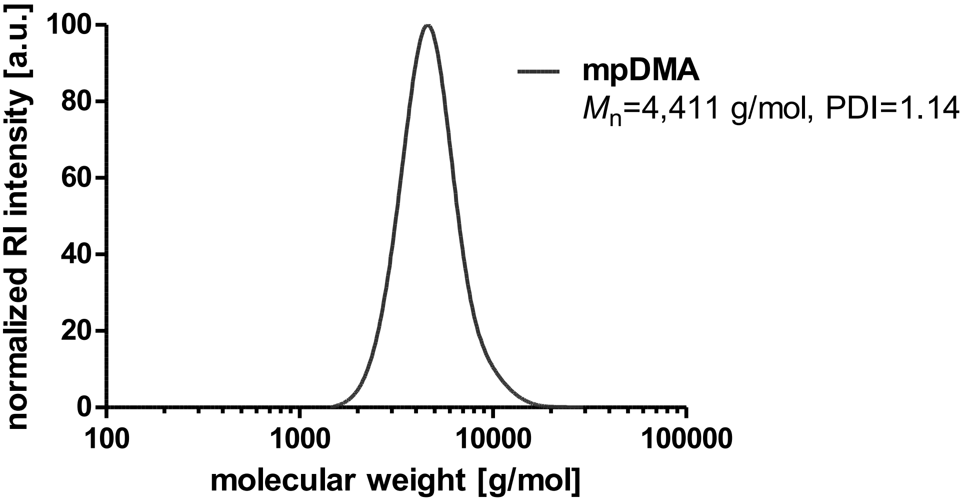

Figure S9: SEC traces of mpDMA with HFIP as eluent.

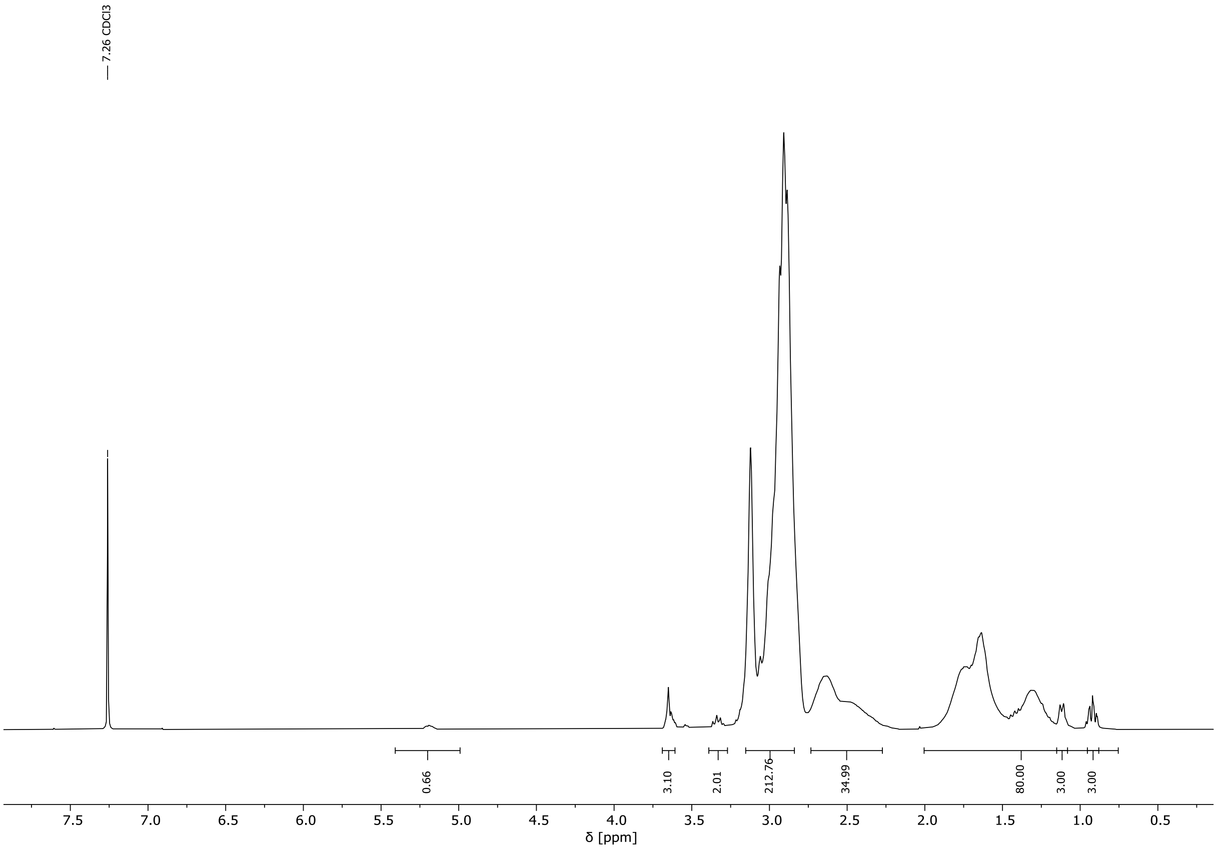

Figure S10: ^{1}H NMR (300 MHz, $CDCl_3$) spectrum of mpDMA.

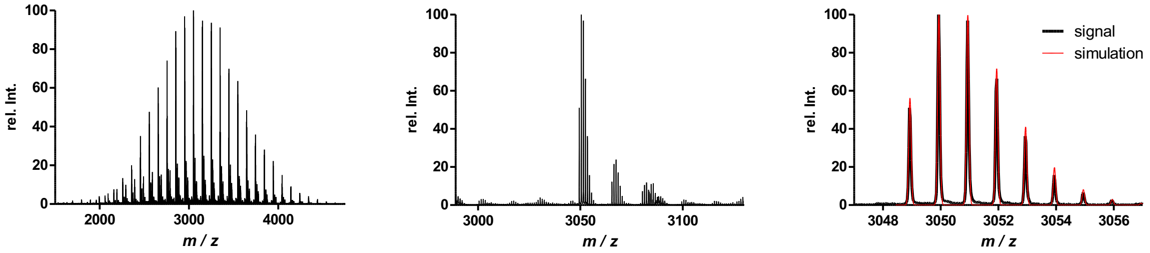

Figure S11: MALDI-ToF MS data of mpDMA. Full polymer mass range (left); zoomed mass range of DP with highest relative intensity (middle); overlay of DP with highest relative intensity and its corresponding simulated isotope pattern (MNa^+; right).

mpDMA-SSC$_2$H$_4$OH via *S*-(2-hydroxyethyl) 4-methylbenzenesulfonothioate

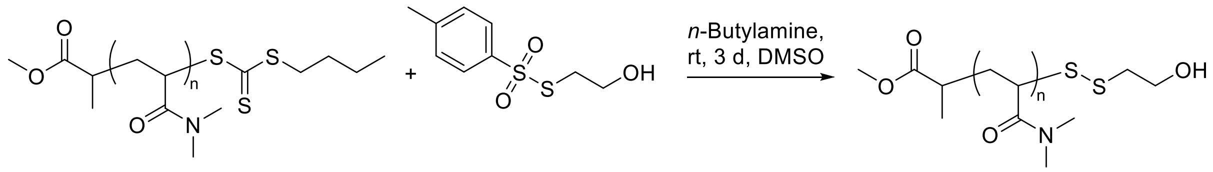

SEC (HFIP; PMMA standard): M_n = 4,478 g mol^{-1}; M_w = 5,110 g mol^{-1}; PDI = 1.14.

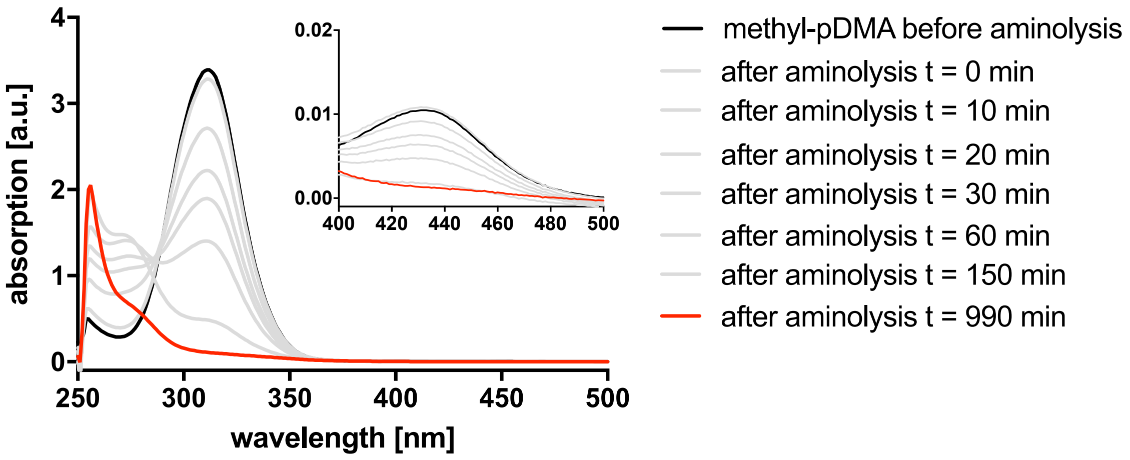

Figure S12: UV/Vis spectroscopy of pDMA after addition of *N*-butylamine. The disappearance of the two absorption maxima at 310 nm and 430 nm indicate a quantitative aminolysis of the trithiocarbonate end group.

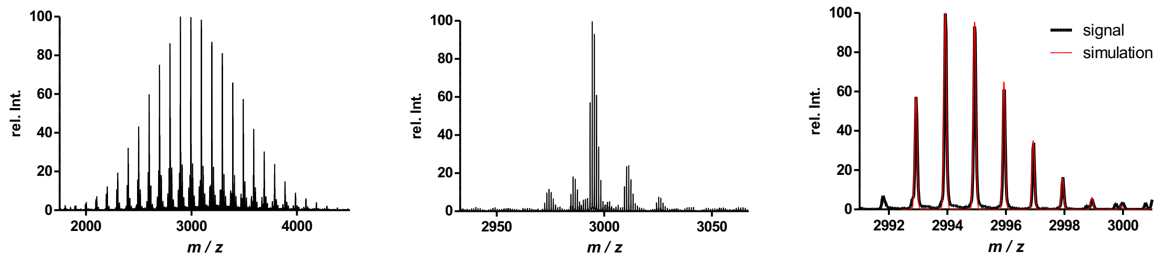

Figure S13: MALDI-ToF MS data of mpDMA-SSC$_2$H$_4$OH via *S*-(2-hydroxyethyl) 4-methylbenzene-sulfonothioate. Full polymer mass range (left); zoomed mass range of DP with highest relative intensity (middle); overlay of DP with highest relative intensity and its corresponding simulated isotope pattern (MNa$^+$; right).

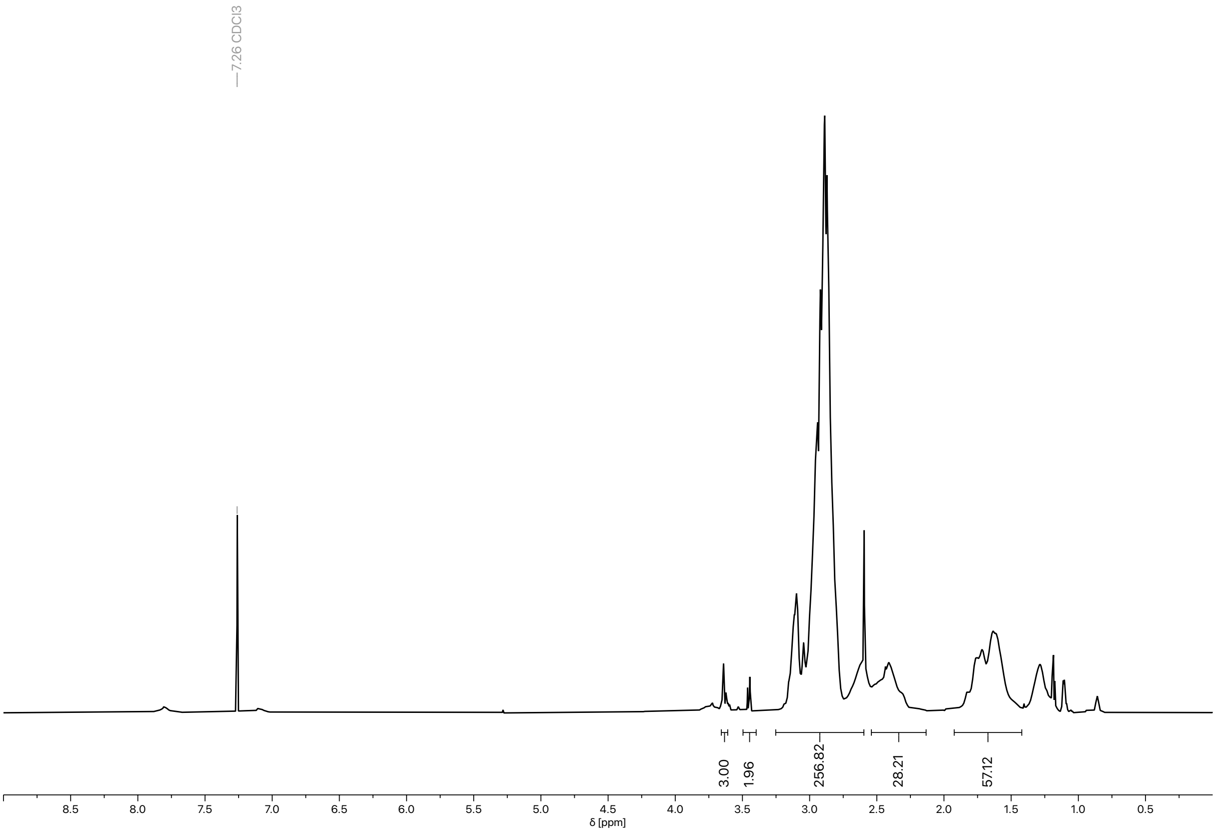

Figure S14: ^{1}H NMR (250 MHz, $CDCl_3$) spectrum of mpDMA-SSC_2H_4OH.

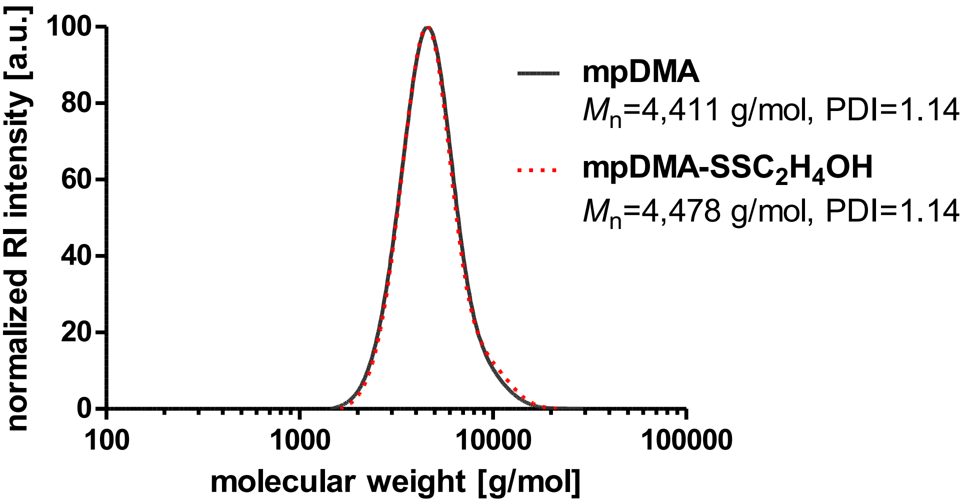

Figure S15: SEC traces of mpDMA and mpDMA-SSC_2H_4OH with HFIP as eluent.

mpDMA-SIL-COPFP

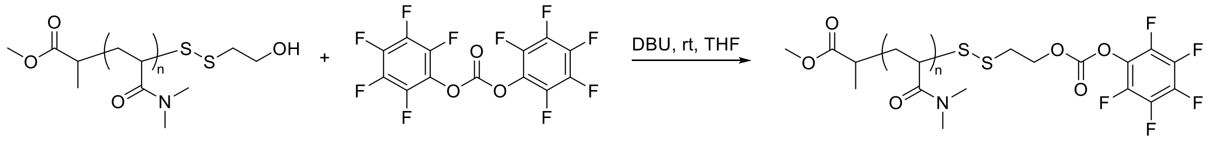

SEC (HFIP; PMMA standard): M_n = 5,195 g mol^{-1}; M_w = 6,124 g mol^{-1}; PDI = 1.18.

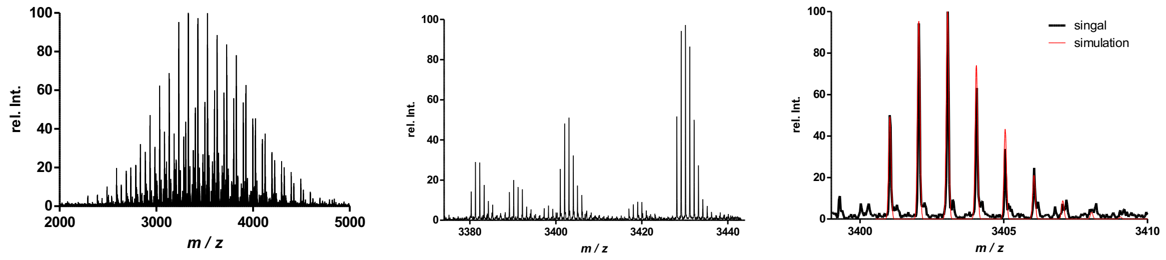

Figure S16: MALDI-ToF MS data of mpDMA-SIL-COPFP. Full polymer mass range (left); zoomed mass range of DP with highest relative intensity and impurities (middle); overlay of DP with highest relative intensity and its corresponding simulated isotope pattern (M+Na$^+$; right).

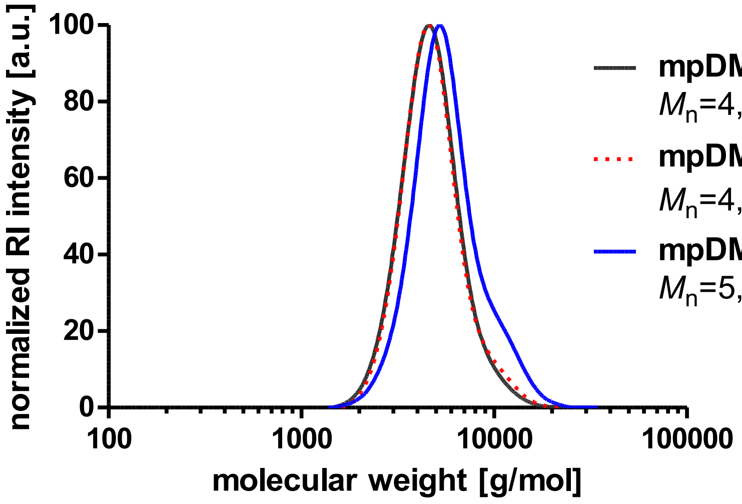

Figure S17: SEC traces of mpDMA, mpDMA-SSC$_2$H$_4$OH and mpDMA-SIL-COPFP with HFIP as eluent.

mpDMA-SIL-IMD

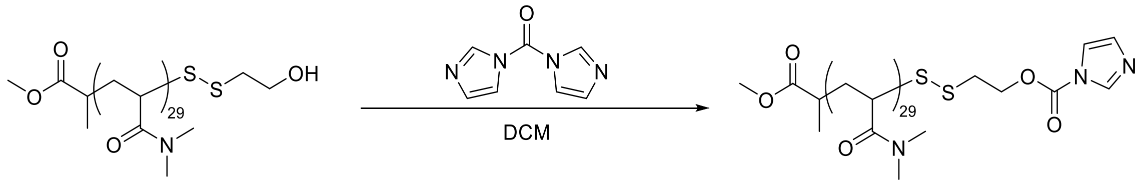

SEC (HFIP; PMMA standard): M_n = 5,346 g mol^{-1}; M_w = 6,466 g mol^{-1}; PDI = 1.21.

^{1}H-NMR (250 MHz, $CDCl_3$) δ [ppm] = 8.17 – 8.10 (m, 1H, IMD-*H*), 7.45 – 7.39 (m, 1H, IMD-*H*), 7.12 – 7.02 (m, 1H, IMD-*H*), 4.66 – 4.55 (m, 2H, –CO–O–C*H*$_2$–), 3.70 – 3.60 (m, 3H, *H*$_3$C–O–), 3.26 – 2.76 (m, 340H, –N–(C*H*$_3$)$_2$), 2.74 – 2.34 (m, 57H, –C*H*–CON(CH$_3$)$_2$), 1.89 – 1.28 (m, 116H, –C*H*$_2$–CH–CONR$_2$ and –S–C*H*$_2$–C$_2$H$_2$–), 1.13 (d, *J* = 6.9 Hz, 3H, –CH–C*H*$_3$).

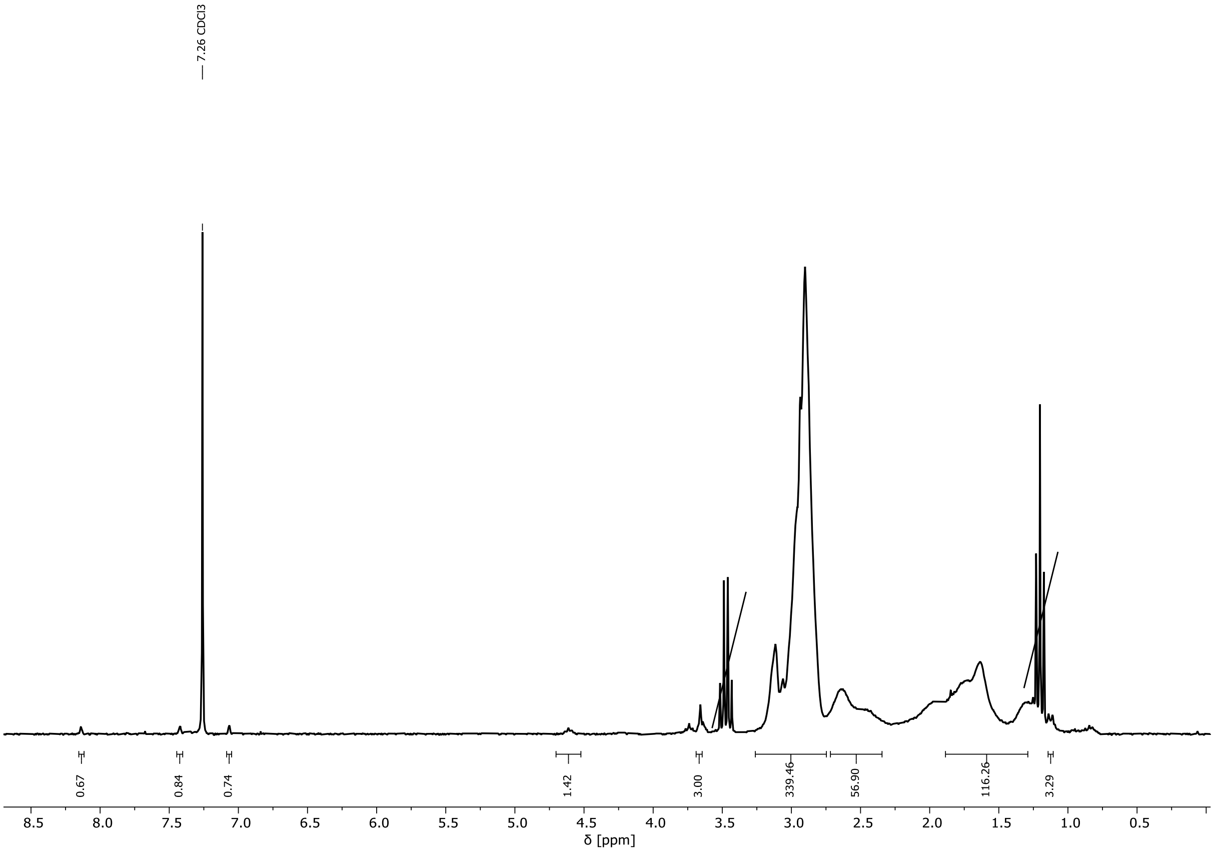

Figure S18: ^{1}H NMR (250 MHz, $CDCl_3$) spectrum of mpDMA-SIL-IMD.

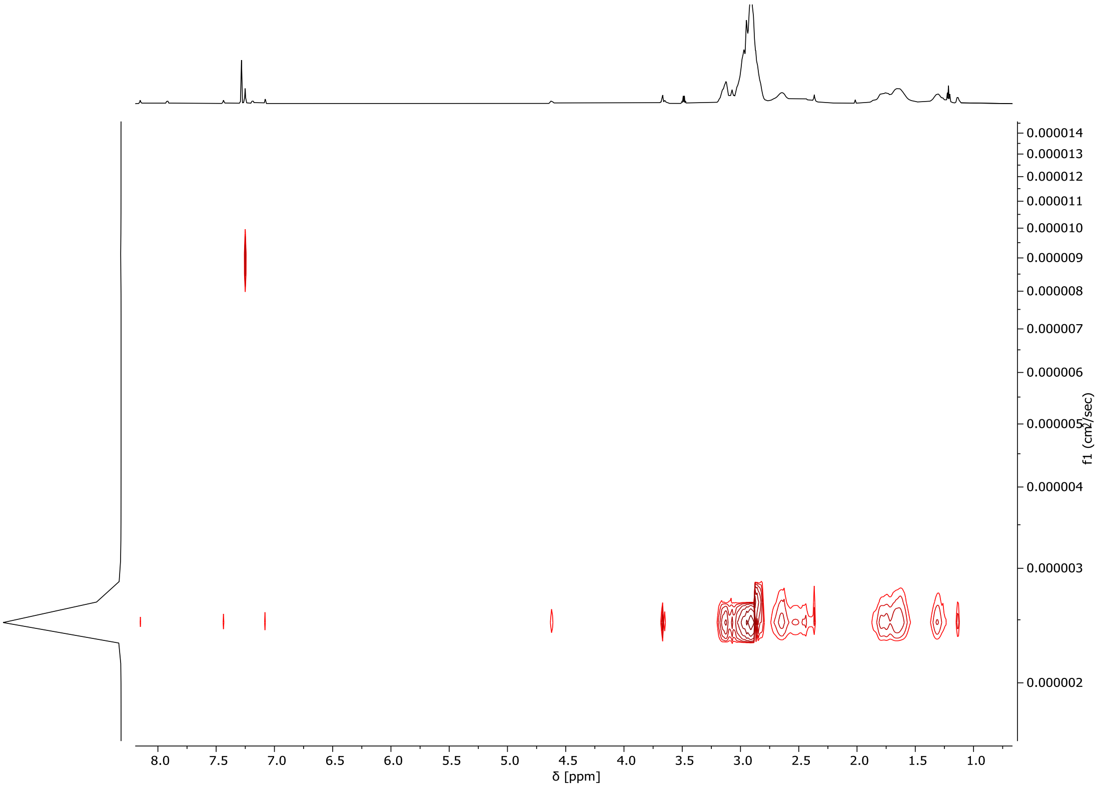

Figure S19: DOSY experiment of mpDMA-SIL-IMD.

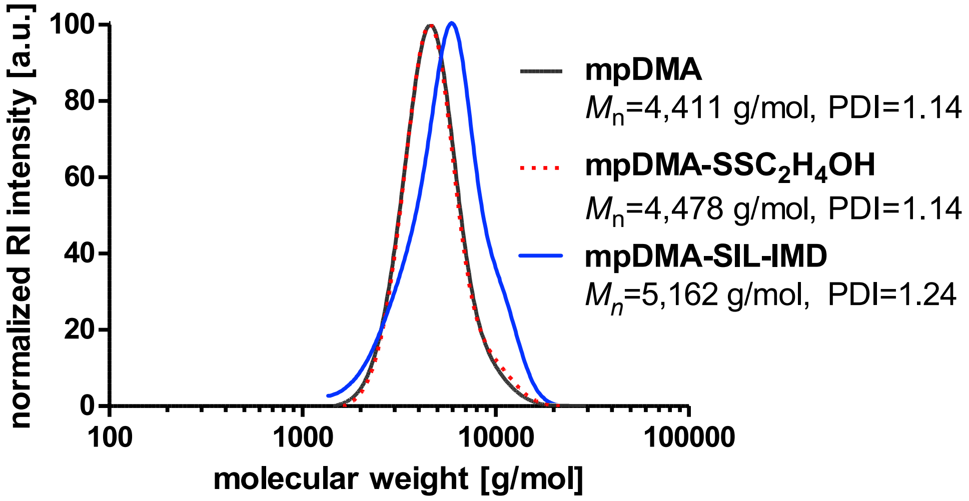

Figure S20: SEC traces of mpDMA, mpDMA-SSC$_2$H$_4$OH and mpDMA-SIL-IMD with HFIP as eluent.

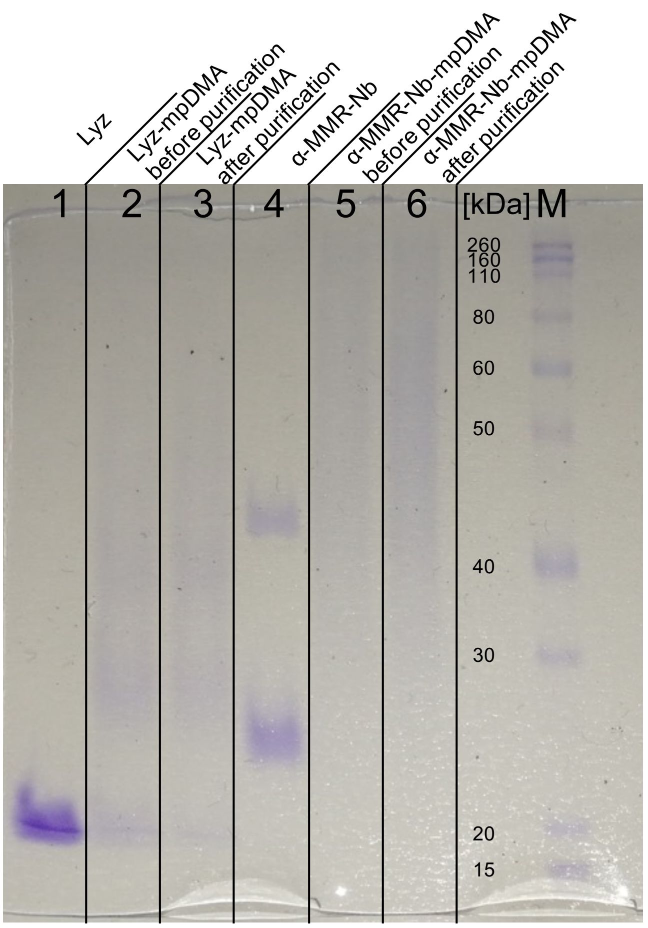

Figure S21: SDS-PAGE of Lyz and α-MMR Nb before and after modification with mpDMA-SIL-IMD, followed by purification using Vivaspin® 500 centrifugal concentrators (molecular weight cutoff 10 kDa).

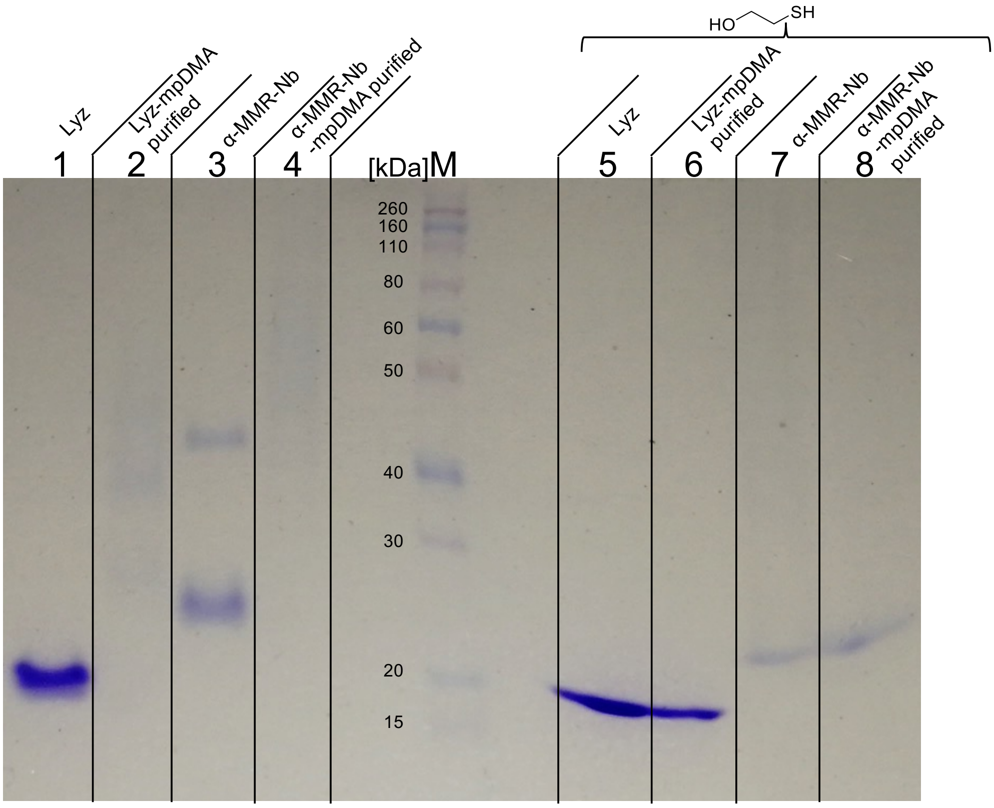

Figure S22: SDS-PAGE of Lyz and α-MMR Nb before and after modification with mpDMA-SIL-IMD and purification using Vivaspin® 500 centrifugal concentrators (molecular weight cutoff 10 kDa). On the right, all samples were additionally treated with mercaptoethanol liberating the unmodified native proteins.

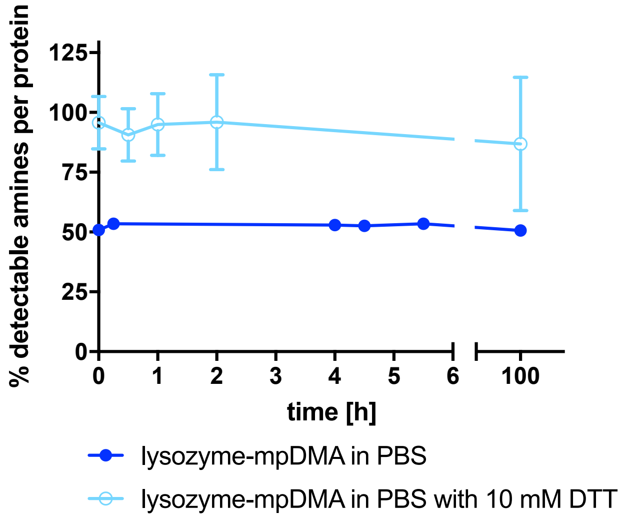

Figure S23: The percentage of detectable amines per protein was determined for lysozyme by fluorescamine assay in PBS with and without 10 mM DTT over time. Note that after exposure to DTT the protein was immediately released quantitatively, while without reducing agents the modified protein remained stable over prolonged time.

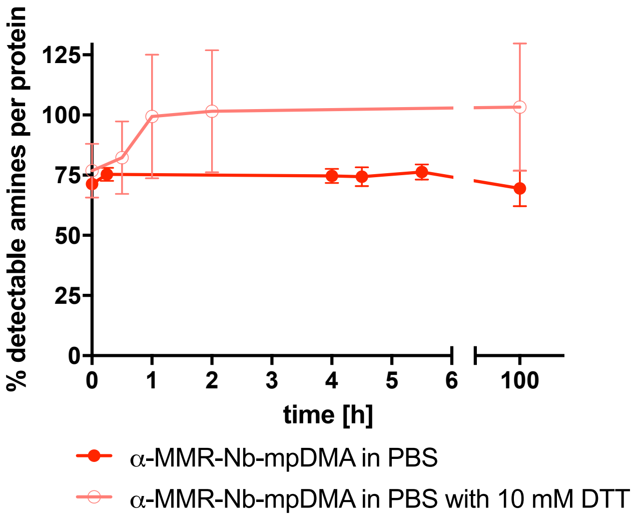

Figure S24: The percentage of detectable amines per protein was determined for α-MMR Nb by fluorescamine assay in PBS with and without 10 mM DTT over time. Note that after exposure to DTT the protein was released only within 1 h quantitatively, while without reducing agent the modified protein remained stable over prolonged time.

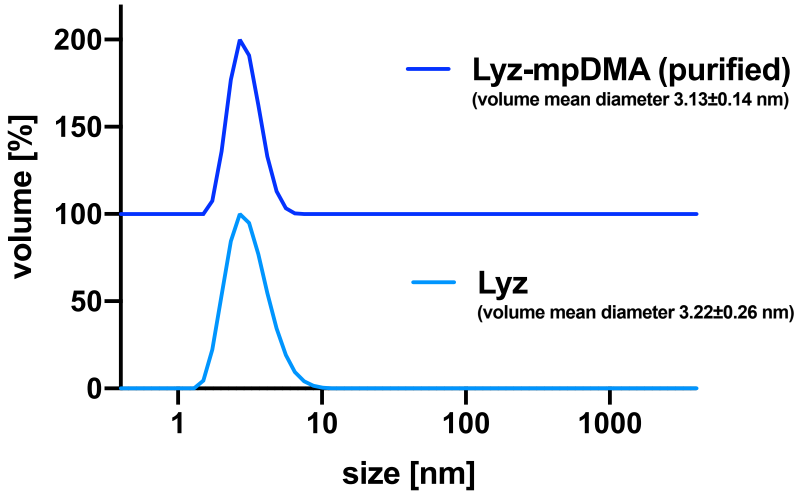

Figure S25: Dynamic light scattering (DLS) size distribution data of lysozyme (Lyz) and polymer-conjugated Lyz-mpDMA showing no significant influence on the protein size due to the small molecular weight of the conjugated mpDMA polymer.

References

(1) Scherger, M.; Räder, H. J.; Nuhn, L. Self-Immolative RAFT-Polymer End Group Modification. *Macromol. Rapid Commun.* **2021**, *42* (8), 2000752. https://doi.org/10.1002/marc.202000752.
